# Fracture-healing effects of Rhizoma Musae ethanolic extract: An integrated study using UHPLC-Q-Exactive-MS/MS, network pharmacology, and molecular docking

**DOI:** 10.1371/journal.pone.0313743

**Published:** 2025-01-14

**Authors:** Jian Zhang, Wanyan Shen, Fanzhi Liu, Hehe He, Shuquan Han, Lina Luo

**Affiliations:** 1 GuiZhou Institute of Subtropical Crops, Guizhou Academy of Agricultural Sciences, Guiyang, China; 2 Research and Development Department, Guizhou Weikang Zifan Pharmaceutical Co., Ltd., Guiyang, China; Airlangga University Faculty of Medicine: Universitas Airlangga Fakultas Kedokteran, INDONESIA

## Abstract

**Background:**

Fracture disrupts the integrity and continuity of the bone, leading to symptoms such as pain, tenderness, swelling, and bruising. Rhizoma Musae is a medicinal material frequently utilized in the Miao ethnic region of Guizhou Province, China. However, its specific mechanism of action in treating fractures remains unknown. This study aimed to elucidate the chemical constituents of the ethanol extract of Rhizoma Musae (EERM) and investigate its fracture-healing mechanism using network pharmacology.

**Methods:**

The chemical profile of EERM was characterized via UHPLC-Q-Exactive-MS/MS. Subsequently, a comprehensive network of compounds, targets, and pathways was constructed using network pharmacology approaches. The interactions between the active compounds of EERM and their targets were validated through molecular docking, molecular dynamics simulation and in vitro cell experiments.

**Results:**

EERM contained 522 identified compounds. Topological analysis of the protein-protein interaction (PPI) network identified 59 core targets, including key proteins like AKT1, IL-6, and EGFR, known for their anti-inflammatory properties and ability to enhance bone cell proliferation and differentiation. Gene Ontology analysis indicated the involvement of EERM in biological processes such as peptidyl-serine phosphorylation, response to xenobiotic stimulus, and nutrient level regulation. KEGG analysis suggested that EERM’s mechanism may involve signaling pathways such as PI3K-Akt, lipid and atherosclerosis, EGFR tyrosine kinase inhibitor resistance, and MAPK pathways. Molecular docking and molecular dynamics simulations results demonstrated a strong binding affinity between the main compounds of EERM and key targets. In vitro cell experiments demonstrate that EERM enhances cell proliferation by upregulating the expression levels of EGFR and STAT3, while simultaneously downregulating AKT1 and CASP3.

**Conclusion:**

This study investigates the potential active compounds of EERM and its key targets in regulating multiple pathways of fracture, leading to promoting bone cell proliferation. These results offer valuable insights for the future development and clinical application of Rhizoma Musae.

## 1. Introduction

The dried rhizome of *Musa basjoo* Sieb. et Zucc., referred to as Rhizoma Musae, is integral to the traditional medicine of the Miao ethnic group in Guizhou Province, China. Recognized for its therapeutic efficacy, Rhizoma Musae is listed in the "Quality Standards of Traditional Chinese and Ethnic Medicinal Materials in Guizhou Province" (2019) [1]. Prior phytochemical analyses have identified numerous compounds in Rhizoma Musae, including volatile oils, phenols, phenalenone, alkaloids, and acenaphthene derivatives [2]. These constituents are renowned for their pharmacological effects, such as anti-inflammatory, antibacterial, and bone formation-promoting properties [3].

The bone is a composite structure composed of cortical bone and trabecular bone, and it contains components such as hydroxyapatite, collagen, a small amount of proteoglycan, non-collagen proteins, and water [4]. Cortical bone provides strength and stability, while trabecular bone offers flexibility and support. Fractures, which disrupt bone integrity and continuity, present symptoms like pain, tenderness, swelling, and bruising, with the possibility of bone deformation and abnormal movements [5]. The risk of fractures varies significantly among different groups, influenced by factors such as fracture type, demographics, age, and obesity [6]. Osteoblasts exist in cortical bone and are mainly generated from bone mesenchymal stem cells. The process of fracture healing requires osteoclasts to remove old or damaged bone, and then osteoblasts replace it with new bone [7]. Osteoblasts also secrete growth factors and cytokines that regulate bone remodeling and repair [8]. Therefore, osteoblasts are important guardians of bone health and play a crucial role in the growth, development, and repair of bones.

Traditional Chinese Medicine (TCM) offers notable benefits in fracture healing, primarily due to its anti-inflammatory [9] and antioxidant properties [10], with a low side effect profile, underscoring its role in fracture management [11]. The Gukang capsule, containing Rhizoma Musae as a principal ingredient, has demonstrated efficacy in treating osteoporosis, fractures, and related injuries [12]. Preliminary studies indicate that the ethanolic extract of Rhizoma Musae (EERM) promotes bone cell growth and differentiation, as evidenced by elevated levels of alkaline phosphatase (ALP), Ca^2+^, and osteocalcin secretion [13]. Nevertheless, the precise molecular mechanisms underpinning these medicinal benefits remain to be elucidated.

Network pharmacology, a burgeoning discipline grounded in systems biology, network analysis of biological systems, and targeted signal node selection for multi-target drug design, is pivotal in elucidating the mechanisms of TCM, thereby facilitating their extensive exploration and clinical application [14]. This study utilized UHPLC-Q-Exactive-MS/MS technology to analyze the chemical compounds of EERM and investigated its mechanism in fracture treatment through network pharmacology and molecular docking. These insights are vital for future clinical trials and the advancement of pharmaceutical research in this domain.

## 2. Materials and methods

### 2.1 Experimental instruments

The primary equipment comprised an ultra-high-pressure liquid chromatograph (Vanquish, Thermo Fisher Scientific, USA), a Q Exactive HFX mass spectrometer (Thermo Fisher Scientific), a UHPLC-Q Exactive HFX, and an ultra-high-performance liquid chromatography-tandem static orbitrap mass spectrometer (Thermo Fisher Scientific), a Centrifuge 5430 R (Eppendorf, Germany), an SCI-VS vortex mixer (Scilogex, USA), an ultrasonic cleaner SB25-12DTD (Ningbo Xinzhi Biotechnology Co., Ltd., China), SpectraMax M5/M5e multiscan spectrum (Molecular Devices, USA), CO2 incubator (Thermo Fisher, USA), and CFX384 Multiple real-time fluorescent quantitative PCR apparatus (Bio-Rad, USA).

### 2.2 Chemical reagents

The study utilized coarse Rhizoma Musae powder provided by Guizhou Weikang Zifan Pharmaceutical Co., Ltd. (batch number YC01-003-20230801). The solvents employed in the extraction and analysis processes included methanol, acetonitrile, formic acid, and isopropanol (all of the analytical grade, Anpu), as well as 95% ethanol (analytical grade) procured from Sangon (Shanghai, China), and ultrapure water. Human Osteoblasts hFOB1.19 (Shanghai Cell Bank, Chinese Academy of Sciences, China). DMEM/F12 (Gibco, USA). Fetal Bovine Serum (Gibco, USA); PBS (Sangon, ShangHai). 0.25% Trypsin-EDTA (Thermo Fisher, USA). TRIzol Plus RNA Purification Kit(Thermo Fisher, USA). RNase-Free DNase Set (Qiagen, Germany). CCK-8 (Tongren Institute of Chemistry, Japan).

### 2.3 Chemical composition analysis of EERM

#### 2.3.1 Sample preparation

During the preparatory phase, 1 g of coarse Rhizoma Musae herb powder was initially immersed in 2 mL of 45% ethanol for 30 minutes to ensure thorough saturation. Subsequently, the sample underwent further extraction in 8 mL of 45% ethanol for 6 hours. After this period, 1 mL of the resulting supernatant was combined with 2 mL of a methanol-acetonitrile (1:1, v/v) solution, vortexed for 60 seconds, and sonicated at a low temperature for 30 minutes. Following sonication, the mixture was centrifuged at 12,000 rpm for 10 minutes at 4°C. The supernatant was then extracted and subjected to chilling at −20°C for 1 hour to precipitate proteins. Another round of centrifugation under the same conditions was performed, after which the supernatant was collected, lyophilized, and reconstituted in 100 μL of 50% acetonitrile. Finally, the vortexed sample was centrifuged again at 12,000 rpm for 10 minutes at 4°C, and the supernatant was collected for analysis.

#### 2.3.2 Analytical conditions

Chromatographic conditions: Liquid chromatography was conducted using a Waters HSS T3 column (100 × 2.1 mm, 1.8 μm), with the column temperature maintained at 40°C to ensure optimal separation. The mobile phase comprised Phase A (0.1% formic acid in water) and Phase B (0.1% formic acid in acetonitrile), with a flow rate of 0.3 mL/min. The elution protocol commenced with 0% Phase B for 1 minute, gradually increasing to 95% Phase B over the next 11 minutes, maintaining 95% Phase B for 1 minute, then swiftly returning to 0% Phase B within 0.1 minutes, and finally holding at 0% Phase B for 3.9 minutes. Samples were preserved at 4°C in an autosampler throughout the analysis, with an injection volume of 2 μL.

Mass spectrometry protocols: Primary and secondary spectral data were acquired using a Q Exactive HFX high-resolution mass spectrometer, utilizing Electrospray Ionization under specific conditions: sheath gas flow at 40 units, auxiliary gas flow at 10 arbitrary units, ion spray voltage set to 3000 V/−2800 V, capillary temperature at 350°C, and ion transfer tube temperature at 320°C. The system, in Full-ms-ddMS2 mode, detected ions in both positive and negative modes across a mass range of 70–1,050 Da, achieving resolutions of 70,000 and 17,500 for primary and secondary scans, respectively. The compounds were characterized based onthe Sanshu Biotech’s proprietary database, specialized in TCM, and their unique secondary mass spectrometry fragmentation pattern matching technique. The relative contents of each compound in EERM were obtained by the area normalization method.

### 2.4 Network pharmacology research

#### 2.4.1 Identification of EERM-related targets

The chemical constituents of EERM and their structures were sourced from the NCBI PubChem database (https://pubchem.ncbi.nlm.nih.gov/). The isomeric SMILES structures of these constituents were uploaded to the Swiss Target Prediction database (http://www.swisstargetprediction.ch/) for target prediction. Additionally, the UniProt database (https://www.uniprot.org/) was utilized to standardize gene nomenclature, thereby aiding in the identification of drug targets associated with EERM.

#### 2.4.2 Identification of fracture targets and construction of protein-protein interaction (PPI) network

To identify fracture-related targets, the term "Fracture" was searched across three genetic databases pertinent to diseases: GeneCards (https://www.genecards.org/), Comparative Toxicogenomics Database (CTD; https://ctdbase.org/), and the Online Mendelian Inheritance in Man (OMIM) database (https://www.omim.org/). The collected data underwent organization and deduplication to create a comprehensive list of fracture-related disease targets.

The online tool Venny (https://bioinfogp.cnb.csic.es/tools/venny/) facilitated the identification of common targets between the active compounds of EERM and known fracture-related targets by intersecting their respective targets. These common targets were further analyzed using the STRING database (https://cn.string-db.org/) with a minimum interaction score of ≥ 0.4. The resultant protein-protein interaction (PPI) network map and TSV format files were subsequently analyzed using Cytoscape software (version 3.9.0) for topological analysis, culminating in a detailed network diagram of "drug compounds-targets-fracture."

#### 2.4.3 Enrichment analyses for GO functions and KEGG pathways

The targets were subjected to Gene Ontology (GO) and Kyoto Encyclopedia of Genes and Genomes (KEGG) enrichment analyses using R software to investigate the potential biological functions and primary signaling pathways implicated in fracture treatment with EERM. The enrichment results were filtered to highlight significant differences and then ranked in descending order based on their P-values, with a significance threshold set at a q-value of < 0.05.

#### 2.4.4 Molecular docking

A greater overlap between disease treatment targets and drug action targets resulted in higher degree values in topological analysis [15]. The top five targets in the PPI network, determined by their degree value, were identified as critical for fracture treatment. Utilizing Amoxicillin as a positive control, these targets underwent molecular docking analysis with the top five core active compounds of EERM, also selected based on degree value. Core target-related protein structures in PDB format were retrieved from the RCSB PDB database (https://www.rcsb.org/), and the active component structures were sourced from the PubChem database. AutoDock Vina 1.1.2 software was utilized for preprocessing target proteins and small molecules, including format transformation and binding site analysis, to validate the docking interactions. Core targets were visually represented using PyMol software.

#### 2.4.5 Molecular dynamics simulation

Gromacs version 2022.03 software was utilized to conduct a 50 ns molecular dynamics (MD) simulation on the receptor protein with the highest binding energy to the ligand molecule identified in the molecular docking results. The CHARMM36 force field was employed for this simulation. The complex resulting from the molecular docking was utilized as the initial structure for the MD simulations, with the small molecule ligands being handled using the Generalized Amber Force Field. The complex was dissolved using three-point transferable intermolecular potential solvents. The solutes were contained within the isothermal isovolumetric ensemble, where the system was gradually heated from 0 K to 300 K. Subsequently, equilibration was achieved in the isothermal isobaric ensemble at a temperature of 300 K and a pressure of 1 Bar.

### 2.5 In vitro cell assays

#### 2.5.1 Cell culture

Once the hFOB1.19 cells were resuscitated, cell passage was performed when the cells reached 80% to 95% confluence. To resuspend the cells, add 0.25% Trypsin-EDTA medium that has been preheated to 37°C. Subsequently, transfer the cells to a new culture dish at a density of 5 × 10^5^ cells/mL and continue the culturing process in a cell incubator maintained at 37°C with 5% CO2.

#### 2.5.2 Cell proliferation assays

Collect the hFOB1.19 cells that have reached the logarithmic phase of growth. The cells were treated with EERM at concentrations of 0, 60, 80, 100, 120, 140, and 160 μg/mL, respectively. For the control group, blank medium was used. The cells were seeded in 96-well plates, with six replicates in each group and 3 × 10^3^ cells per well. After 48 hours of incubation, 10 μL of CCK-8 reagent was added to each well, followed by an additional incubation period of 1.5 hours. The absorbance value at 450 nm was measured using a microplate reader to calculate the cell proliferation inhibition rate for each experimental group.

#### 2.5.3 Quantitative real-time polymerase chain reaction (qPCR)

RNA extraction from the cells was performed utilizing an RNA extraction kit, which was subsequently followed by the synthesis of cDNA. The PCR primers were created with the help of Primer Premier 6.0 and were synthesized by Sangon Biotech (Shanghai). The sequences of the primers can be found in Table 1. The conditions for PCR amplification were set at 95°C for 30 seconds, succeeded by 40 cycles of 95°C for 5 seconds and 60°C for 30 seconds. The internal reference gene used was β-actin, and the relative mRNA expression levels were determined employing the 2−△△Ct method.

**Table 1:** Primers sequences.

Gene name	Forward primer	Reverse primer
β-actin	GATGACCCAGATCATGTTTGAGAC	GGAGTCCATCACGATGCCAGT
AKT1	GCCCCACTTCCCCCAGTTCT	CCGCCTCTCCATCCCTCCAA
IL-6	CCTTCGGTCCAGTTGCCTTCT	GTGTGGGGCGGCTACATCTTT
EGFR	AGGGCCTTGCCGCAAAGTGT	CCACCGGCAGGATGTGGAGAT
STAT3	CTCCAGGCACCTTCCTGCTAA	GGGTCTTACCGCTGATGTCCTT
CASP3	CAGAACTGGACTGTGGCATTG	GATGAACCAGGAGCCATCCTT

#### 2.5.4 Statistical analysis

The experimental results were analyzed using GraphPad Prism version 10.1.2. All results are presented as mean ± SEM. An independent unpaired two-tailed t-test was employed to compare the two groups.

## 3. Results

### 3.1 Identification of EERM compounds

**Fig 1:**
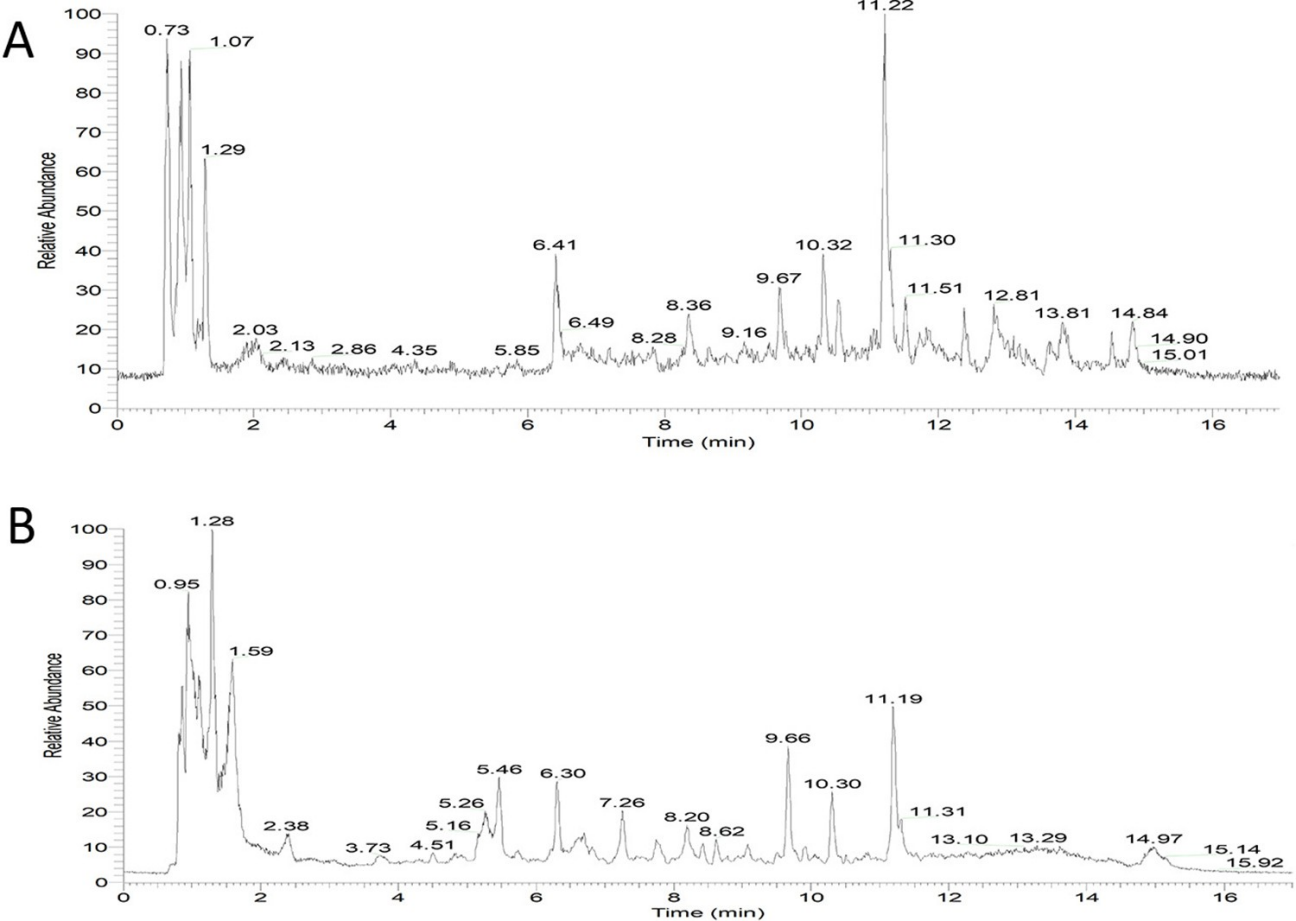
Total ion chromatogram of active compounds from EERM in positive ion (A) and negative ion (B) modes.

The study of EERM cataloged 522 compounds, as detailed in **S1 Table**, including 288 identified in positive ion mode and 234 in negative ion mode (**Fig 1**). These compounds were classified by chemical nature into the following categories: 62 flavonoids, 53 prenol lipids, 44 organooxygen compounds, 34 carboxylic acids and derivatives, 27 coumarins and derivatives, 24 fatty acyls, 20 benzene and substituted derivatives, 18 isoflavonoids, 16 cinnamic acids and derivatives, 12 phenols, 11 benzopyrans, 7 purine nucleosides, and 194 others.

### 3.2 Acquisition of potential targets for EERM in fracture treatment

**Fig 2:**
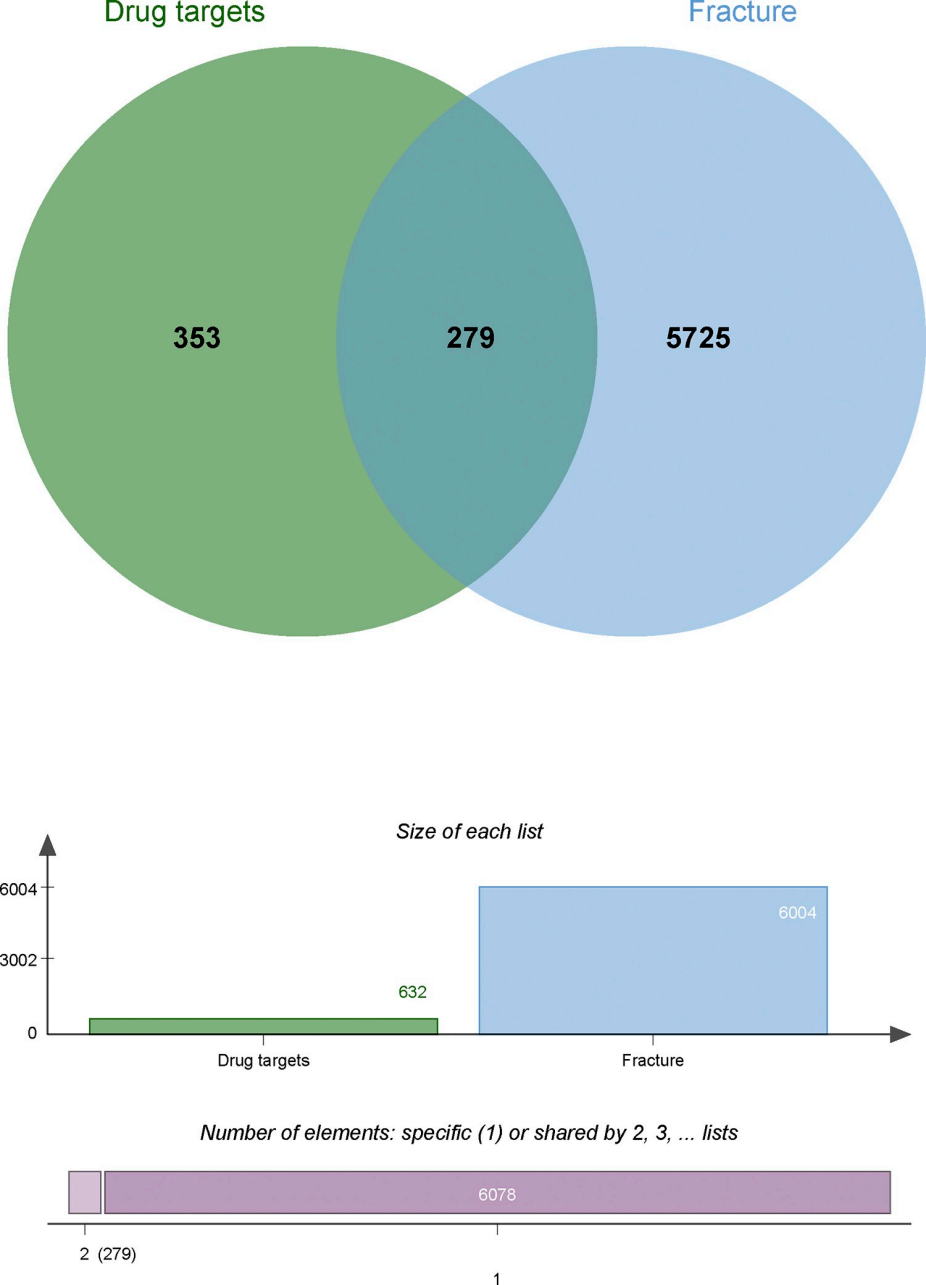
Venn diagram illustrating the intersected targets of EERM compounds and fracture.

The top 70 compounds in EERM, selected based on their relative peak area (**Table 2**), were analyzed using the Swiss Target Prediction database, identifying 632 potential medicinal targets. Subsequent research into GeneCards, CTD, and OMIM databases identified 1,546, 5,732, and 654 fracture-related targets, respectively. Intersecting these results with drug target genes revealed 279 potential targets for fracture treatment (Fig 2).

**Table 2:** Relative content analysis of the top 70 compounds found in EERM.

No.	Compound	Formula	Rt(min)	m/z	Ion mode	PubChem ID
1	Betaine	C_5_H_11_NO_2_	0.94	118.0864	POS	247
2	alpha-Isowighteone	C_20_H_18_O_5_	12.08	303.1009	POS	91885205
3	Cholinesulfuric acid.	C_5_H_13_NO_4_S	0.93	184.0636	POS	485
4	3-O-Caffeoylquinic acid	C_16_H_18_O_9_	5.75	353.0874	NEG	1794427
5	Vanillic acid	C_8_H_8_O_4_	7.24	167.0341	NEG	8468
6	4-Hydroxybenzoic acid	C_7_H_6_O_3_	6.70	137.0233	NEG	135
7	Bisdemethoxycurcumin	C_19_H_16_O_4_	12.06	309.1114	POS	5315472
8	_7_beta-Hydroxyrutaecarpine	C_18_H_12_N_3_O_2_	12.87	303.1010	POS	15225951
9	Eleutherazine B	C_22_H_36_N_4_O_6_	7.22	453.2698	POS	20839739
10	Ginsenoside Rg_1_	C_42_H_72_O_14_	10.54	823.4803	POS	441923
11	5-Methoxypiperonal	C_9_H_8_O_4_	13.35	181.0494	POS	22016
12	ADENOSINE	C_10_H_13_N_5_O_4_	4.06	268.1035	POS	60961
13	Carnitine	C_7_H_15_NO_3_	0.94	162.1123	POS	288
14	2,16-Kauranediol 2-O-beta-D-allopyranoside	C_26_H_44_O_7_	12.43	491.2970	POS	73554066
15	Vanillin	C_8_H_8_O_3_	7.76	151.0390	NEG	1183
16	Scopoletin	C_10_H_8_O_4_	8.09	237.0400	NEG_116	5280460
17	1-(3,4-Dihydroxyphenyl)-7-(4-hydroxyphenyl)-4-hept	C_19_H_20_O_4_	11.18	295.1322	POS	11570978
18	Trigonelline	C_7_H_7_NO_2_	1.01	138.0549	POS	5570
19	D-mannitol	C_6_H_14_O_6_	0.93	181.0709	NEG	6251
20	Hydroxyanigorufone	C_19_H_12_O_3_	11.91	287.0710	NEG	11471752
21	myo-Inositol	C_6_H_12_O_6_	1.06	179.0552	NEG	892
22	4’-Hydroxy-2-O-methylanigorufone	C_20_H_14_O_3_	12.10	301.0866	NEG	71358480
23	Magnaldehyde B	C_18_H_16_O_3_	12.29	245.0957	POS	5320888
24	L(+)-Ascorbic acid	C_6_H_8_O_6_	1.23	221.0297	NEG	54670067
25	Mercaptobenzothiazole	C_7_H_5_NS_2_	9.90	167.9935	POS	697993
26	3,4-Dihydroxyphenylacetic acid	C_8_H_8_O_4_	5.09	167.0341	NEG	547
27	D-Glutamic acid	C_5_H_9_NO_4_	0.93	148.0603	POS	23327
28	Suavioside A	C_26_H_44_O_8_	11.54	507.2922	POS	73821014
29	Sugeroside	C_26_H_42_O_8_	11.74	505.2764	POS	3082543
30	4-Hydroxyphenylpyruvic acid	C_9_H_8_O_4_	5.85	163.0389	POS	979
31	3-Hexen-1-ol O-b-D-glucopyranoside	C_12_H_22_O_6_	9.25	261.1340	NEG	5318045
32	4-Hydroxycinnamamide	C_9_H_9_NO_2_	6.18	198.0320	NEG	16637983
33	Xylitol	C_5_H_12_O_5_	0.94	151.0601	NEG	6912
34	URIDINE	C_9_H_12_N_2_O_6_	2.79	243.0618	NEG	6029
35	D-LEUCINE	C_6_H_13_NO_2_	2.01	132.1019	POS	439524
36	linoleic acid	C_18_H_32_O_2_	14.89	313.2729	POS	5280450
37	D-Arabinose	C_5_H_10_O_5_	0.95	133.0495	POS	854
38	Opuntiol	C_7_H_8_O_4_	1.06	174.0760	POS	10034839
39	Dehydroacerogenin C	C_19_H_18_O_3_	11.52	259.1113	POS	154790969
40	D-Glucosamine	C_6_H_13_NO_5_	0.96	180.0864	POS	439213
41	caffeic acid	C_9_H_8_O_4_	6.80	163.0389	POS	689043
42	Lucidone	C_15_H_12_O_4_	9.51	301.0714	NEG	11253859
43	D-proline	C_5_H_9_NO_2_	1.03	116.0708	POS	8988
44	Guanosine	C_10_H_13_N_5_O_5_	2.05	284.0983	POS	135398635
45	Hexylitaconic acid	C_11_H_18_O_4_	11.67	213.1126	NEG	11447214
46	13-Hydroxygermacrone	C_15_H_22_O_2_	12.96	235.1689	POS	10399140
47	Aconine	C_25_H_41_NO_9_	12.44	517.3130	POS	20054813
48	Danshenol C	C_21_H_20_O_4_	11.52	301.1216	POS	11688609
49	7,8-Dihydroxycoumarin	C_9_H_6_O_4_	7.13	223.0243	NEG	5280569
50	2-Hydroxypalmitic acid	C_16_H_32_O_3_	14.74	271.2275	NEG	92836
51	Picrotoxinin	C_15_H_16_O_6_	9.54	273.0766	NEG	442292
52	D-Valine	C_5_H_11_NO_2_	0.94	235.1648	POS	71563
53	Notoginsenoside R_1_	C_47_H_80_O_18_	10.22	955.5221	POS	131752529
54	Giffonin R	C_19_H_16_O_3_	10.74	257.0956	POS	134715258
55	Quinic acid	C_7_H_12_O_6_	3.84	173.0446	NEG	6508
56	4-Epialyxialactone	C_10_H_16_O_4_	10.08	199.0969	NEG	14194344
57	Gallic acid	C_7_H_6_O_5_	3.11	169.0133	NEG	370
58	Demethoxycurcumin	C_20_H_18_O_5_	12.09	339.1220	POS	5469424
59	Stemonidine	C_19_H_29_NO_5_	13.29	332.1863	NEG	24721470
60	1,6-anhydro-b-D-Glucose	C_6_H_10_O_5_	1.23	207.0503	NEG	2724705
61	5,7-Dihydroxyphthalide	C_8_H_6_O_4_	6.15	211.0242	NEG	11062751
62	1-Phenyl-2-propanol	C_9_H_12_O	12.38	119.0857	POS	94185
63	Dihydrosesamin	C_20_H_20_O_6_	9.30	355.1183	NEG	10871980
64	Isoderrone	C_20_H_16_O_5_	13.26	317.0813	NEG	14237660
65	7,8-Benzoflavone	C_19_H_12_O_2_	10.78	317.0815	NEG	11790
66	Syringolin A	C_24_H_39_N_5_O_6_	12.18	535.3238	POS	42601513
67	Gamabufotalin	C_24_H_34_O_5_	12.24	403.2450	POS	259803
68	D-Glucosaminic acid	C_6_H_13_NO_6_	0.98	160.0603	POS	73563
69	7-Deoxyechinosporin	C_10_H_9_NO_4_	6.96	190.0497	POS	11745823
70	Methyl gallate	C_8_H_8_O_5_	6.19	165.0184	NEG	7428

### 3.3 Analysis of the PPI network

**Fig 3:**
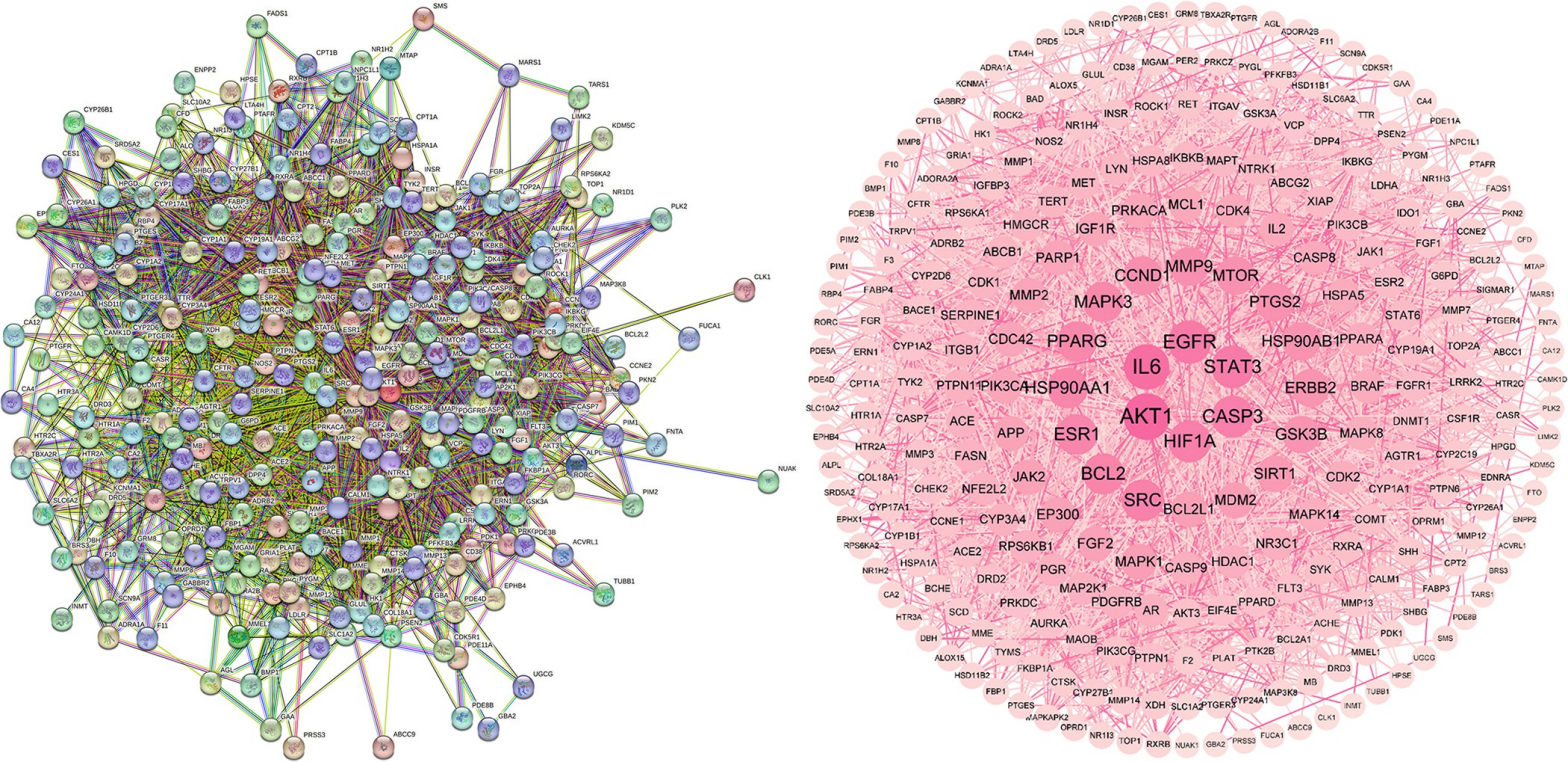
Visualization of the protein-protein interaction (PPI) network of potential targets for EERM.

To investigate the interaction relationships among the 279 potential targets, these were uploaded to the STRING database, unveiling a PPI network consisting of 278 proteins and 4,788 interactions (**Fig 3**). Key targets were identified by applying criteria where "Degree, betweenness centrality, and closeness centrality exceeded average values," resulting in 59 core targets (**S2 Table**). The top 10 targets, determined by degree value (**Table 3**)—AKT1, IL-6, epidermal growth factor receptor (EGFR), STAT3, CASP3, HIF1A, SRC, BCL2, ESR1, and HSP90AA1—demonstrate that EERM compounds predominantly influence these core targets, thereby affecting their positive impact on fractures.

**Table 3:** Topological parameter analysis of the top 10 core targets.

Targets	Degree	BC	CC
AKT1	163	0.060431	0.701266
IL6	153	0.066853	0.683951
EGFR	137	0.035427	0.659524
STAT3	133	0.029540	0.656398
CASP3	128	0.021801	0.642691
HIF1A	127	0.028595	0.642691
SRC	125	0.045036	0.639723
BCL2	121	0.016691	0.630979
ESR1	119	0.026902	0.626697
HSP90AA1	118	0.032546	0.630979

Note: BC, betweenness centrality. CC, betweenness centrality.

### 3.4 Development of the "EERM-Fracture" network diagram

**Fig 4:**
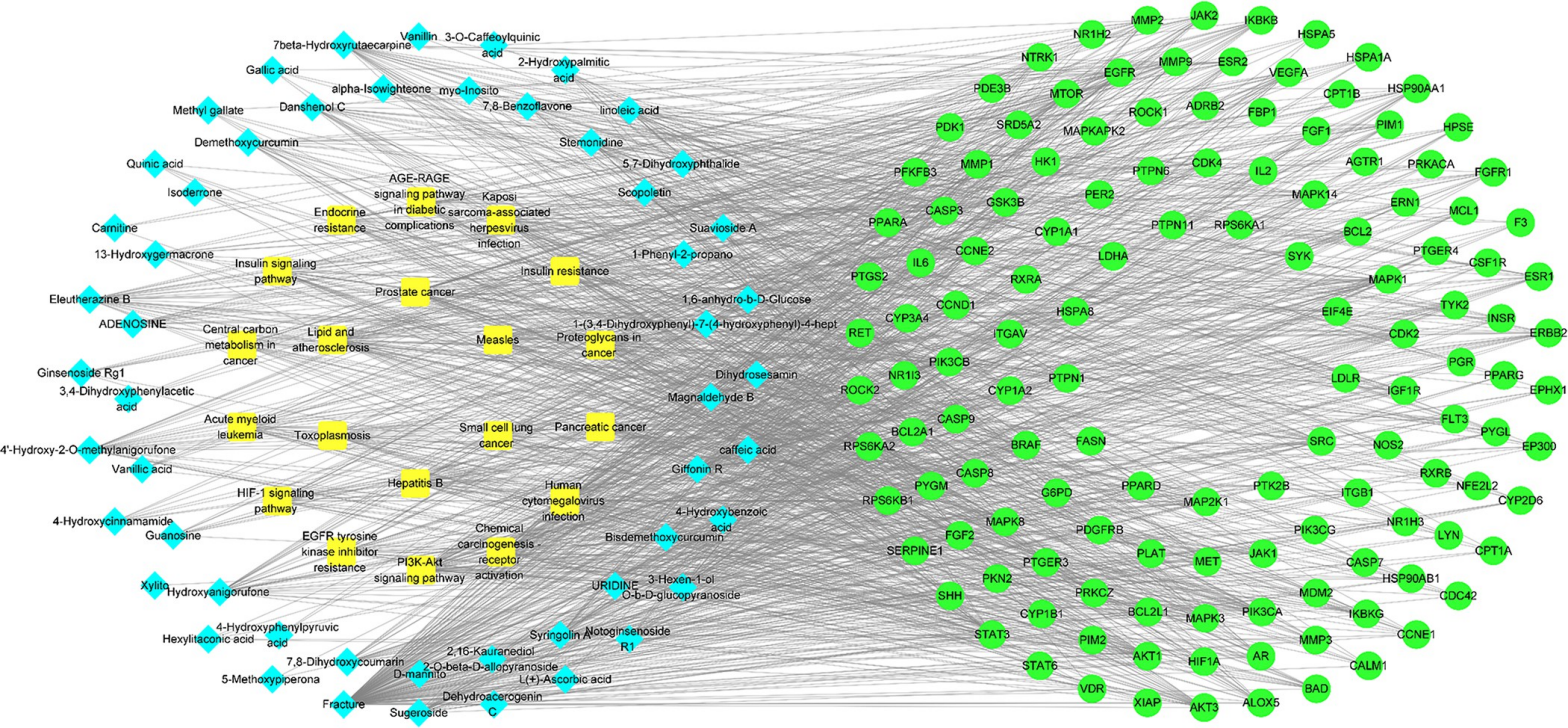
Schematic representation of the "Component-target-pathway" network illustrating the mechanism of EERM. Drug compounds are depicted as blue diamonds, targets are shown as green circles, and signaling pathways are indicated as yellow squares.

The "EERM-Fracture" network diagram (**Fig 4**) was constructed using Cytoscape software (version 3.9.0), with the Network Analyzer plugin employed for topological parameter analysis. The analysis revealed an average adjacency of 6.383 nodes, network heterogeneity of 2.712, a network density of 0.02, and a centrality measure of 0.849. Nodes with higher degree values were identified as central nodes, with the five most active compounds being 4′-hydroxy-2-O-methylanigorufone (degree = 57), Syringolin A (degree = 54), 7β-Hydroxyrutaecarpine (degree = 53), linoleic acid (degree = 51), and Hydroxyanigorufone (degree = 50; **Table 4**), highlighting their critical roles within the network.

**Table 4:** Top 10 active compounds in EERM with potential efficacy against fractures.

No.	Compound	Degree	BC	CC
1	4’-Hydroxy-2-O-methylanigorufone	57	0.018168	0.386826
2	Syringolin A	54	0.016453	0.382249
3	7beta-Hydroxyrutaecarpine	53	0.016280	0.381346
4	linoleic acid	51	0.014990	0.377778
5	Hydroxyanigorufone	50	0.013473	0.378664
6	Sugeroside	49	0.013132	0.373410
7	Eleutherazine B	48	0.013197	0.376896
8	Giffonin R	47	0.010489	0.370838
9	Bisdemethoxycurcumin	40	0.009961	0.371692
10	ADENOSINE	29	0.005923	0.364149

### 3.5 GO analysis and KEGG pathway enrichment analysis

**Fig 5:**
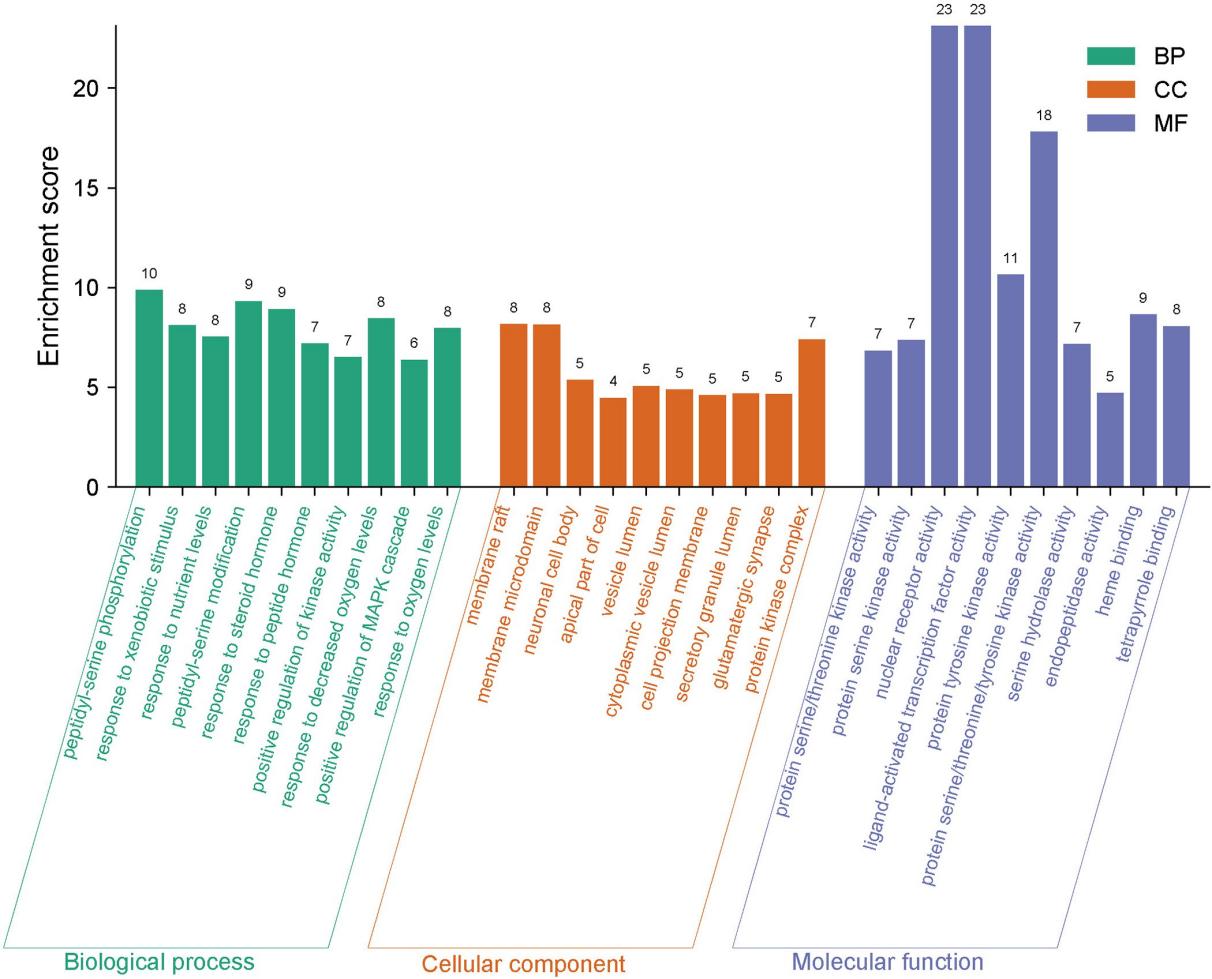
Gene Ontology functional enrichment analysis results for the potential targets of EERM.

The "Cluster Profiler" package in R software facilitated the GO enrichment analysis of 279 potential targets, resulting in the identification of 3,266 entries (**Fig 5**). Within these, the biological process (BP) category comprised 2,900 entries, primarily involving peptidyl-serine phosphorylation, response to xenobiotic stimulus, response to nutrient levels, peptidyl-serine modification, and response to steroid hormone. The cellular component (CC) category included 136 entries, predominantly associated with the membrane raft, membrane microdomain, neuronal cell body, apical part of the cell, and vesicle lumen. The molecular function (MF) category featured 230 entries, focusing on protein serine/threonine kinase activity, protein serine kinase activity, nuclear receptor activity, ligand-activated transcription factor activity, and protein tyrosine kinase activity.

**Fig 6:**
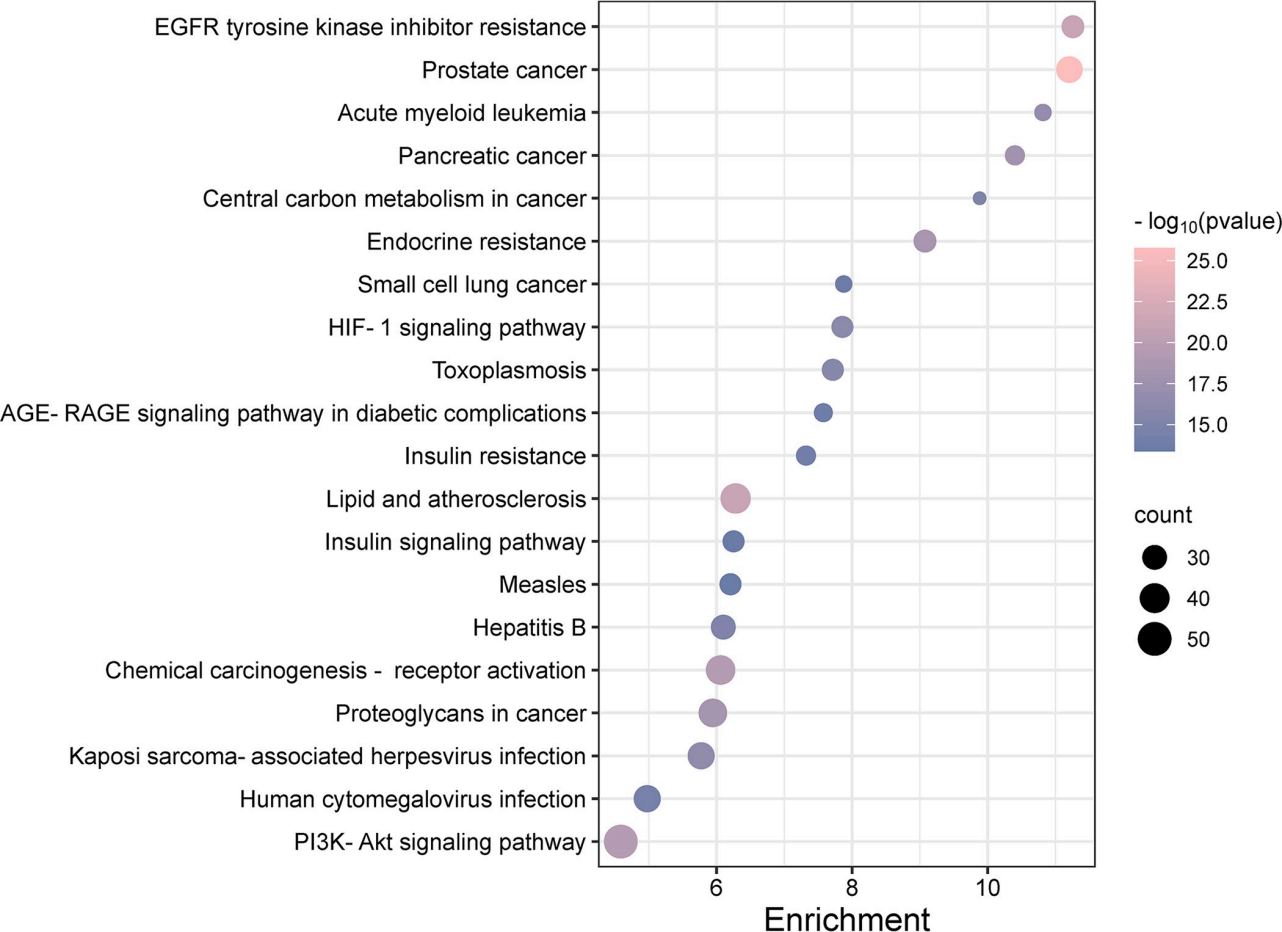
KEGG pathway enrichment analysis of EERM targets.

Subsequent KEGG pathway enrichment analysis on 279 potential targets, conducted using the "Cluster Profiler" package in R, identified 169 significant signaling pathways (q-value < 0.05; **Fig 6**). This analysis notably highlighted pathways related to prostate cancer, lipid metabolism and atherosclerosis, resistance to EGFR tyrosine kinase inhibitors (TKIs), the PI3K-Akt signaling pathway, chemical carcinogenesis *via* receptor activation, endocrine resistance, the role of proteoglycans in cancer, pancreatic cancer, acute myeloid leukemia, and infections caused by Kaposi sarcoma-associated herpesvirus.

### 3.6 Molecular docking findings

**Fig 7:**
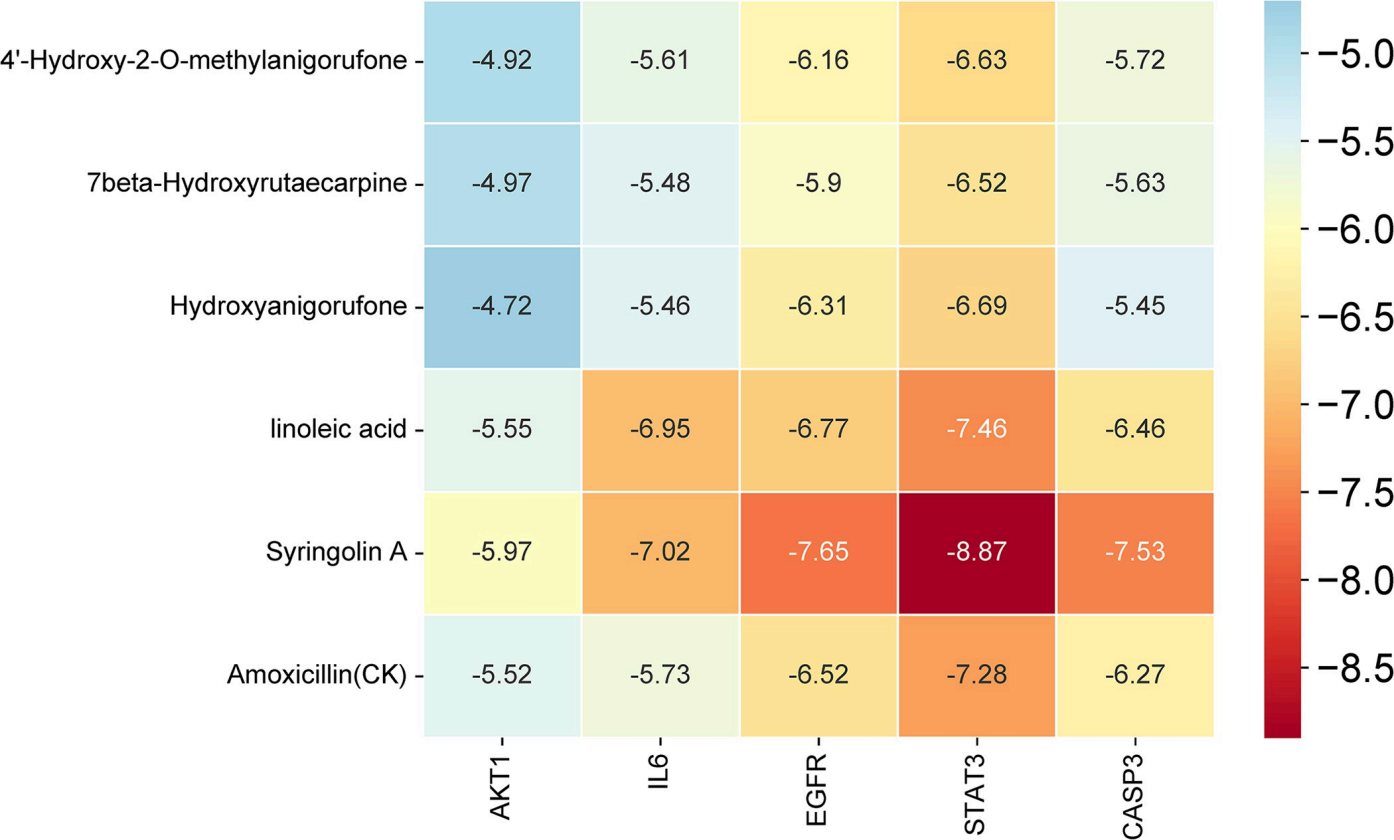
Heat map illustrating the binding energies between EERM compounds and their targets.

Utilizing Amoxicillin as a positive control, molecular docking was conducted between the initial five core compounds and the first five identified targets, with the subsequent calculation of binding energy. Binding energies below zero indicated the spontaneous binding capability of drug compounds to target proteins. Specifically, binding energies below −5 kcal/mol demonstrated substantial binding affinity, while those below −7.0 kcal/mol indicated strong binding capacity [16]. Molecular docking results (**Fig 7**) revealed that key compounds and targets could spontaneously bind, with 21 pairs exhibiting binding energies below −5 kcal/mol and 5 pairs below −7.0 kcal/mol. Specially, Inoleic acid and Syringolin A exhibit stronger binding affinities to AKT1, CASP3, EGFR, IL6, and STAT3 compared to the positive control Amoxicillin.

**Fig 8:**
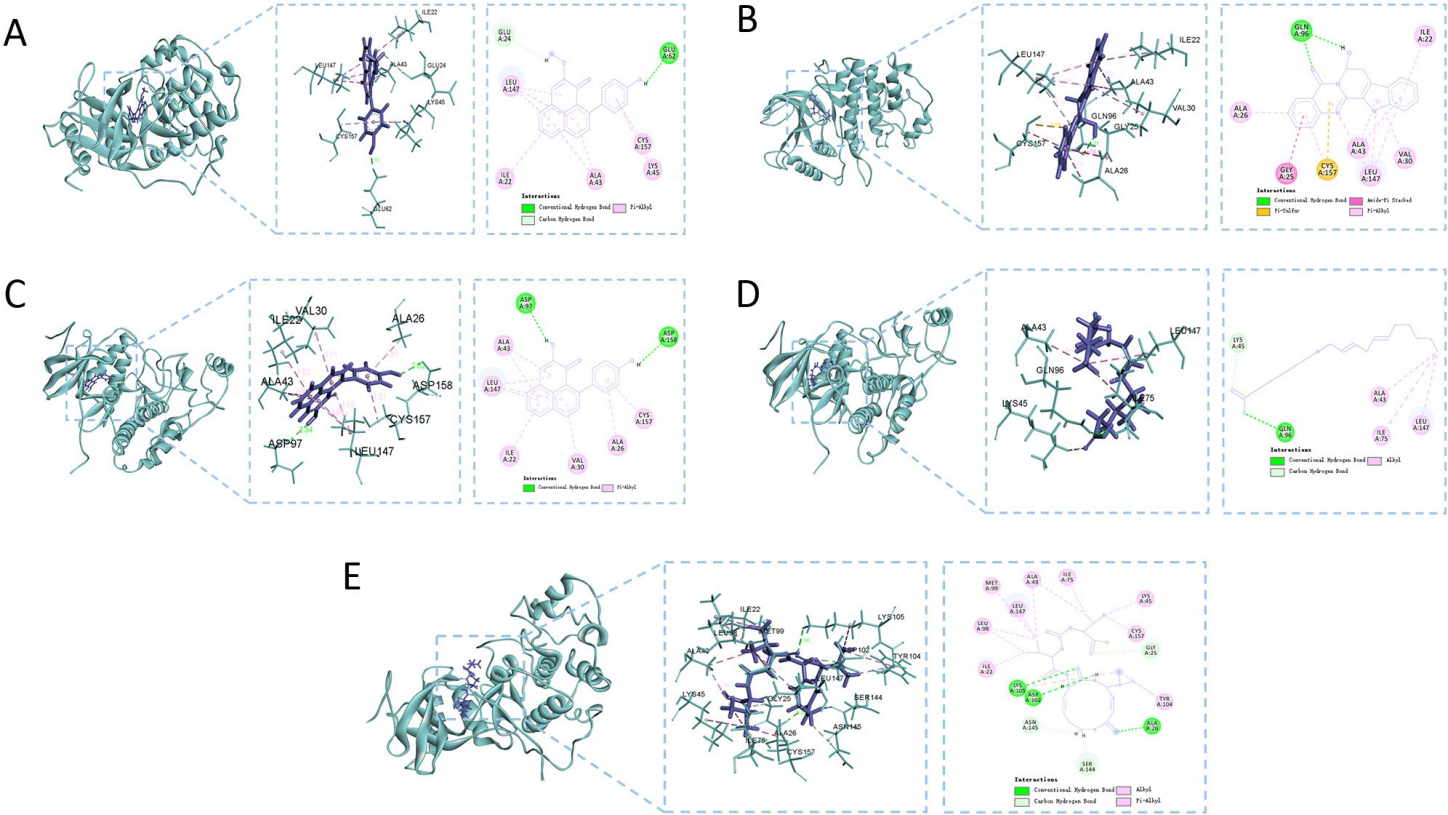
Molecular docking diagrams. (A) 4-hydroxy-2-O-methylanigorufone and STAT3; (B) 7β-hydroxyrutaecarpine and STAT3; (C) hydroxyanigorufone and STAT3; (D) linoleic acid and STAT3; (E) Syringolin A and STAT3.

Visual analysis of the targets with the highest binding affinities to the core compounds was conducted, as presented in Fig 8. Notably, 4-hydroxy-2-O-methylanigorufone formed a hydrogen bond with STAT3 at GLU A:62, resulting in a binding energy of −6.63 kcal/mol, indicative of strong binding affinity. Similarly, 7β-hydroxyrutaecarpine and STAT3 formed a hydrogen bond at GLN A:96, with a binding energy of −6.52 kcal/mol, suggesting strong binding capacity. Hydroxyanigorufone interacted with STAT3 through hydrogen bonds at ASP A:97 and ASP A:158, with a binding energy of −6.69 kcal/mol, indicating strong binding affinity. Linoleic acid formed a hydrogen bond with STAT3 at GLN A:96, leading to a binding energy of −7.46 kcal/mol, showing very strong binding affinity. Lastly, Syringolin A formed hydrogen bonds with STAT3 at LYS A:105, ASP A:102, and ALA A:26, with a binding energy of −8.87 kcal/mol, demonstrating a highly strong binding affinity.

### 3.7 Molecular dynamics simulation

**Fig 9:**
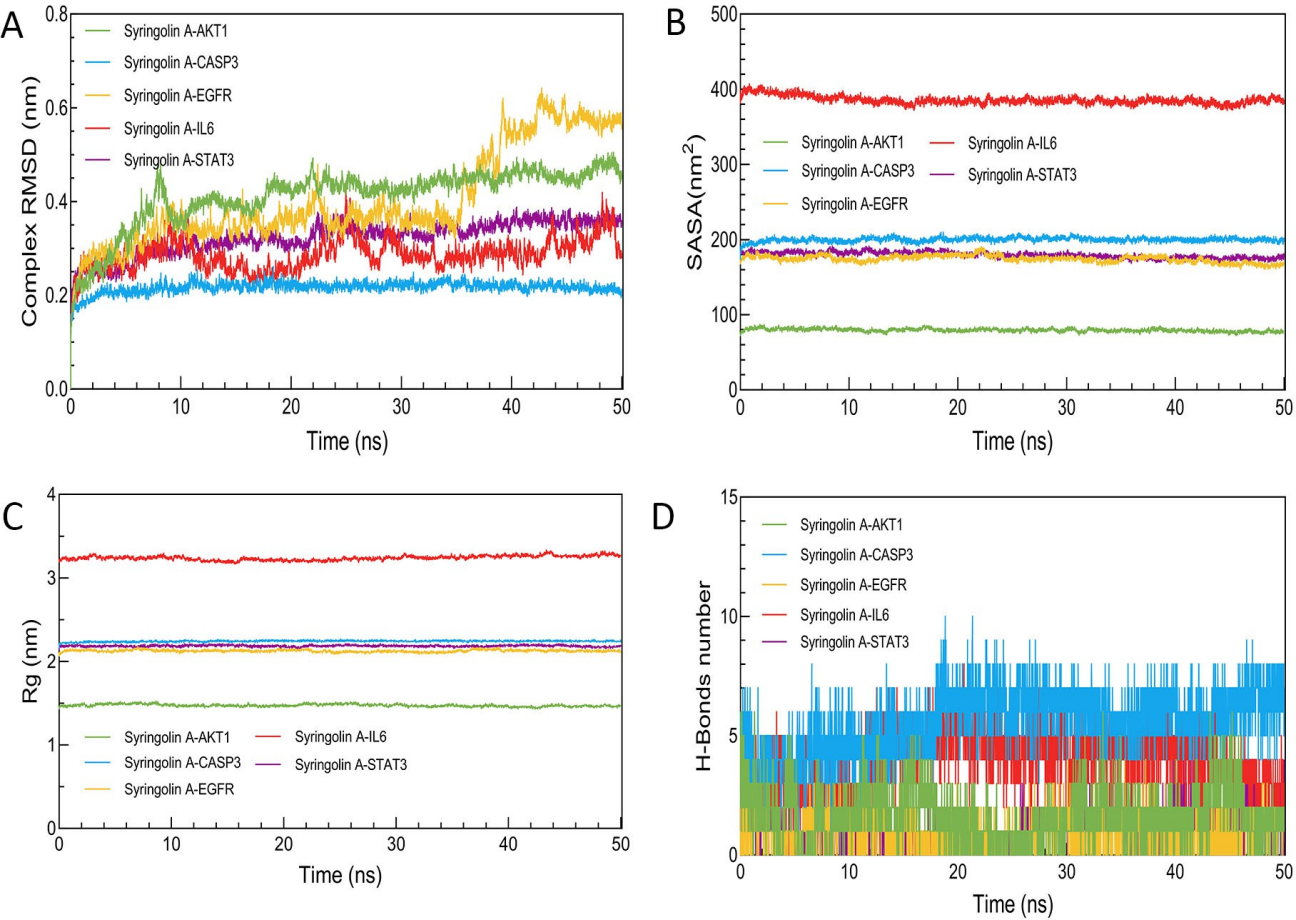
The MD simulation of Syringolin A with AKT1, CASP3, EGFR, IL6, and STAT3 complex systems. (A) RMSD curve. (B) SASA curve. (C) Rg curve. (D) H-bonds number.

This study performed a 50 ns MD simulation analysis on the Syringolin A interactions with AKT1, CASP3, EGFR, IL6, and STAT3 complex systems, based on the molecular docking results. Root mean square deviation (RMSD) is used to assess the fluctuation index of protein conformation. A smaller RMSD value indicates a higher similarity between two structures [17]. The stability of the four Syringolin A complexes with AKT1, CASP3, IL6, and STAT3 gradually improved after 10 ns, as depicted in Fig 9A. However, the RMSD curve for the Syringolin A-EGFR complex fluctuated between 0.35 nm and 0.75 nm at 34 ns, suggesting insufficient stability in the complex conformation. Fig 9B and 9C show that in all simulated systems, the Solvent Accessible Surface Area (SASA) and Radius of Gyration (Rg) remained relatively stable, indicating a close interaction between Syringolin A and the key target. The analysis presented in Fig 9D illustrates the varying number of hydrogen bonds in the Syringolin A-AKT1, Syringolin A-CASP3, Syringolin A-EGFR, Syringolin A-IL6, and Syringolin A-STAT3 complexes over a 50 ns simulation period. The number of hydrogen bonds ranged from 1 to 6 in these complexes, with values of 4–10, 1–4, 3–8, and 1–4 observed for Syringolin A-AKT1, Syringolin A-CASP3, Syringolin A-EGFR, and Syringolin A-STAT3, respectively. Notably, the Syringolin A-CASP3 and Syringolin A-IL6 complexes exhibited a higher propensity to form hydrogen bonds throughout the simulation.

**Fig 10:**
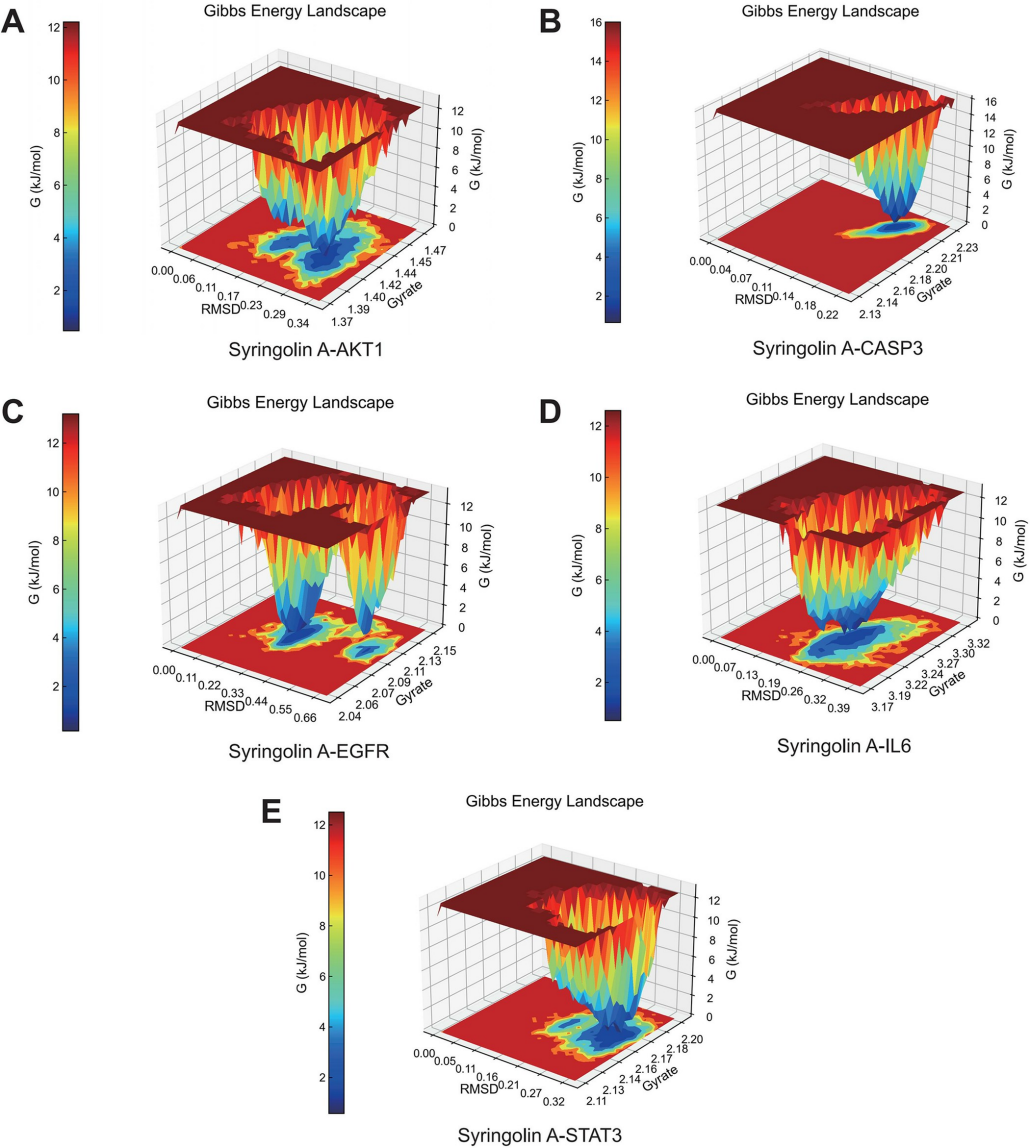
Gibbs energy analysis of five groups of complexes. (A) Syringolin A-AKT1 complex. (B) Syringolin A-CASP3 complex. (C) Syringolin A-EGFR complex. (D) Syringolin A-IL6 complex. (E) Syringolin A-STAT3 complex.

The Gibbs energy landscape of five groups of complexes is illustrated in Fig 10. Within this landscape, there are two distinct energy clusters present in the Gibbs energy 3D topography of the Syringolin A-AKT1, Syringolin A-EGFR, Syringolin A-IL6, and Syringolin A-STAT3 complexes. Conversely, the Gibbs energy 3D topography of the Syringolin A-CASP3 complex displays a single and smooth energy cluster. These findings indicate that all five groups of complexes exhibit the ability to spontaneously combine and possess relatively stable binding capabilities, with the Syringolin A-CASP3 complex demonstrating the strongest binding ability.

The final 20 ns stable RMSD trajectory was calculated using the analysis of protein ligand Molecular Mechanics Generalized Born Surface Area method (MM/PBSA) to determine the complex binding free energy. Table 5 displays the total binding free energy values for different complexes: -17.55 kJ/mol for Syringolin A-AKT1, -38.98 kJ/mol for Syringolin A-CASP3, -17.55 kJ/mol for Syringolin A-EGFR, -21.42 kJ/mol for Syringolin A-IL6, and -31.71 kJ/mol for Syringolin A-STAT3. The results align with the molecular docking findings, showing that all five groups of complexes are capable of binding spontaneously.

**Table 5:** Binding free energy and energy compounds calculated by MM/PBSA (KJ/mol).

Compounds	ΔE_vdw_	ΔE_elec_	ΔG_gas_	ΔG_solvation_	ΔG_Bind_
Syringolin A-AKT1	-23.86	-45.92	-69.78	52.23	-17.55
Syringolin A-CASP3	-46.28	-21.60	-67.88	28.90	-38.98
Syringolin A-EGFR	-34.37	-27.41	-61.78	40.36	-21.42
Syringolin A-IL6	-47.82	-30.20	-78.03	49.81	-28.22
Syringolin A-STAT3	-48.42	-19.64	-68.06	36.35	-31.71

Note: ΔE_vdw_, van der Waals energy. ΔE_elec,_ electrostatic energy. ΔG_gas,_ Gas phase energy. ΔG_solvation,_ Free energy of solvation. ΔG_Bind,_ Total bingding free energy.

### 3.8 EERM promotes the proliferation of hFOB1.19 cells

**Fig 11:**
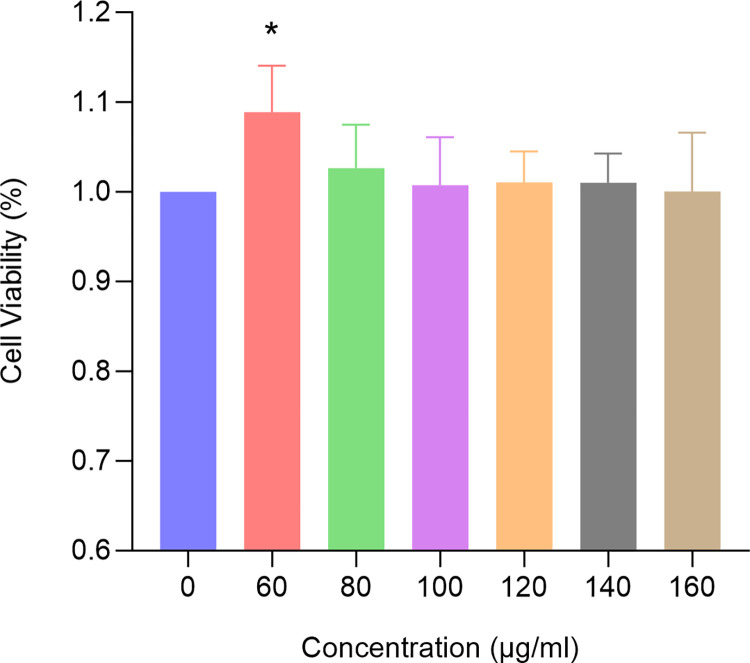
EERM promotes the proliferation of hFOB1.19 cells (x ± s, n = 6). * represents *p* < 0.05.

After 48 hours of treatment with EERM, the proliferation rate of hFOB1.19 cells varied according to the concentration of EERM (Fig 11). Treatment with 60 μg/mL EERM resulted in the highest proliferation rate of hFOB1.19 cells, which was significantly greater than that of the control group (*p* < 0.05).

### 3.9 EERM regulates the expression of related genes

**Fig 12:**
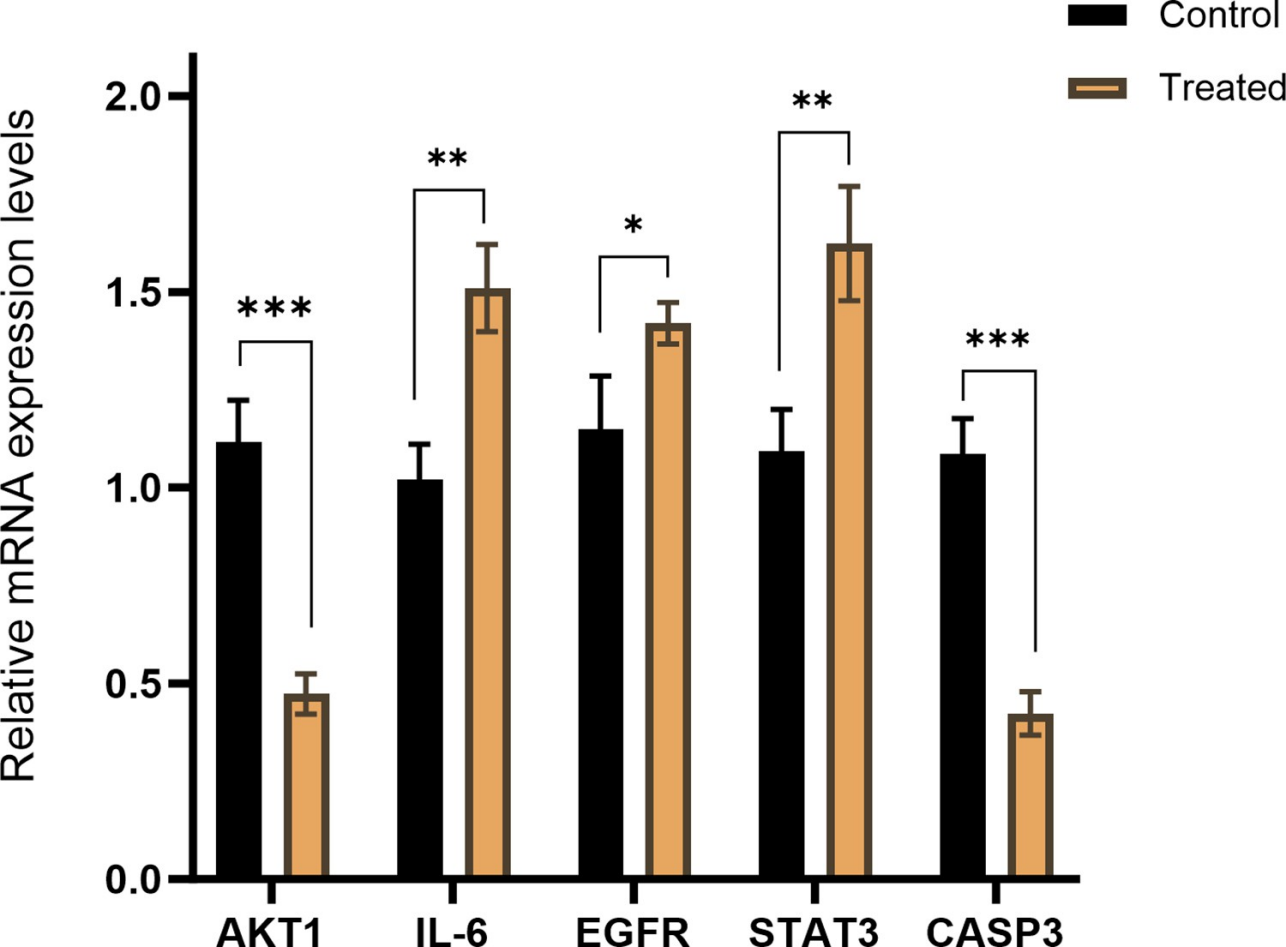
RM decoction regulates the related genes expression in hFOB1.19 cells. (x ± s, n = 9). * represents *p* < 0.05, ** represents *p* < 0.01, *** represents *p* < 0.001.

The expression of related genes in hFOB1.19 cells was analyzed using qPCR technology. Treatment with 60 μg/mL EERM for 48 hours significantly altered the expression levels of these genes (Fig 12). Specifically, EERM markedly up-regulated the expression of EGFR, IL-6, and STAT3, while down-regulating the expression of AKT1 and CASP3.

## 4. Discussion

Rhizoma Musae, a traditional medicine from the Miao ethnic region in Guizhou Province, China, is recognized for its heat-clearing and detoxifying properties [18]. The role and mechanism of Rhizoma Musae in treating fractures are not fully understood. In this study, UHPLC-Q-Exactive-MS/MS technology was used to identify 522 chemical compounds in EERM, with a focus on flavonoids, prenol lipids, and organooxygen compounds. A detailed component-target correlation analysis of the 70 most abundant compounds revealed key compounds effective for fracture treatment.

Hydroxy-2-O-methylanigorufone, a phytoalexin extracted from Musa spp. (Baxijiao) flowers [19], exhibits a notable α-glucosidase inhibitory effect. Syringolin A, produced by the plant pathogen *Pseudomonas syringae* pv. *syringae*, inhibits proteasomal activity in multiple myeloma [20]. Additionally, 7β-hydroxyrutaecarpine, derived from evodiacarpine, is an indopyridine-quinazoline alkaloid that undergoes hydroxylation and was initially detected in the callus tissue of *Phellodendron* [21]. Evodiacarpine is known for its analgesic, anti-inflammatory, and anti-diarrheal pharmacological effects [22]. Linoleic acids, polyunsaturated fatty acids commonly found in the human diet [23], are associated with reduced risks of atherosclerosis, hypertension, and diabetes [24], enhanced immune function [25], and improved musculoskeletal health [26]. Hydroxyanigorufone, a natural compound from banana fruit, plays a key role in phytoalexin synthesis [27].

Network pharmacology, which examines interactions between biomolecules and targets through drug structure and effect similarities [28], provides a comprehensive approach to understanding the mechanisms of TCM and their formulations. This methodology offers new insights into the interaction networks of TCMs in treating complex diseases [29]. The PPI network analysis for fracture treatment using EERM identified 59 key targets, including AKT1, IL-6, EGFR, STAT3, and CASP3. AKT1, encoded by the PKB gene and belonging to the AKT serine/threonine kinase family, plays a crucial role in the PI3K signaling pathway. It affects downstream effectors that are key in abnormal bone cell proliferation, synovial inflammation, as well as osteoclast formation and differentiation [30]. IL-6, a multifunctional cytokine, impacts the immune and nervous systems and is linked to antimicrobial molecule production and cytokine activity [31]. EGFR, a member of the ERBB family of tyrosine kinase receptors, regulates cellular processes such as proliferation, differentiation, division, survival, and oncogenesis [32]. STAT3, part of the STAT family, governs cell growth, differentiation, survival, anti-inflammatory activity, tissue repair, and cancer development [33]. CASP3, from the caspase family of cysteine-dependent aspartate-directed proteases, plays a pivotal role in programmed cell death mechanisms [34] like apoptosis and pyroptosis, crucial in inflammatory diseases [35]. In vitro cell experiments demonstrated that EERM enhanced cell proliferation by upregulating EGFR and STAT3 while downregulating the expression levels of AKT1 and CASP3. These findings are consistent with the gene functions reported in the aforementioned literature.

GO analysis on 279 potential targets revealed associations with various CCs, such as membrane rafts, membrane microdomains, and neuronal cell bodies, all crucial for fracture healing [36]. These findings suggest that these targets could serve as potential therapeutic targets. Additionally, these targets are involved in multiple MFs such as protein serine/threonine kinase activity, protein serine kinase activity, and nuclear receptor activity, affecting BPs like peptidyl-serine phosphorylation, response to xenobiotic stimulus, and nutrient levels. Therefore, EERM’s therapeutic effects on fractures result from the combined action of multiple biological processes.

The KEGG pathway enrichment analysis highlighted 20 key signaling pathways significantly influenced by EERM, including the PI3K-Akt signaling pathway, lipid metabolism and atherosclerosis, and EGFR TKI resistance. The PI3K-AKT pathway, critical for processes such as proliferation, differentiation, invasion, and apoptosis [37], was underscored with 50 core gene targets including GSK3B, IGF1R, and INSR. The analysis also identified inflammation-related targets within lipid and atherosclerosis pathways, such as STAT3, MMP9, MMP1, and AKT1, emphasizing the role of chronic inflammation in atherosclerosis development [38]. EGFR, a transmembrane tyrosine kinase that interacts with EGF family ligands, activates downstream pathways like MAPK, enhancing DNA synthesis and cellular proliferation [39]. TKIs, which inhibit tyrosine kinase phosphorylation and activation, are used in cancer treatment despite common resistance issues [40]. The MAPK pathway, involving JNK, ERK, and p38, is essential for apoptosis, differentiation, and proliferation, primarily through the activation of transcription factors [41]. This approach leverages interconnected, cooperative, and multi-level regulation. EERM achieves therapeutic effects on fractures by targeting key elements, regulating the entire signaling network, and influencing bone cell proliferation and differentiation. However, this research still has some limitations. Additional pharmacological experiments as well as in vitro and in vivo experiments are needed to validate the key targets and associated signaling pathways identified through network pharmacology analysis.

## 5. Conclusion

This study, leveraging UHPLC-Q-Exactive-MS/MS technology and network pharmacology, identified the potential therapeutic effects of EERM on fractures. Molecular docking was employed to simulate and confirm the interactions between key compounds and targets, elucidating the pharmacological basis, critical targets, and pathways involved in treating fractures with EERM. A total of 522 compounds were detected in EERM, with compounds such as 4′-hydroxy-2-O-methylanigorufone, Syringolin A, 7β-hydroxyrutaecarpine, linoleic acid, and hydroxyanigorufone highlighted for their fracture treatment potential. Network pharmacology analysis identified essential targets, including AKT1, IL-6, EGFR, STAT3, and CASP3. EERM promotes cell proliferation by upregulating the expression levels of EGFR and STAT3, while downregulating AKT1 and CASP3. These targets may enhance bone cell proliferation through various potential influencing pathways, including the PI3K-Akt signaling pathway, lipid metabolism and atherosclerosis, EGFR TKI resistance, and the MAPK signaling pathway.

## Supporting information

S1 Table A total of 522 compounds in ethanol extracts from Rhizoma Musae. (DOCX)

S2 Table Analysis of the topological parameters of the core targets. (DOCX)

S1 Graphical abstract (PDF)
