# Supplementary material for: Fracture-healing effects of Rhizoma Musae ethanolic extract: An integrated study using UHPLC-Q-Exactive-MS/MS, network pharmacology, and molecular docking
Source: PLoS One. 2025 Jan 14;20(1):e0313743. doi: 10.1371/journal.pone.0313743 (PMC11731732; doi:10.1371/journal.pone.0313743)
Supplement: S1 Table — (DOCX) [file pone.0313743.s001.docx]

**S1 Table. A total of 522 compounds in ethanol extracts from Rhizoma Musae.**

| No. | Compound | Formula | Rt(min) | m/z | Ion mode | Pubchem ID |
| --- | --- | --- | --- | --- | --- | --- |
| 1 | Betaine | C_5_H_11_NO_2_ | 0.94 | 118.0864 | POS | 247 |
| 2 | alpha-Isowighteone | C_20_H_18_O_5_ | 12.08 | 303.1009 | POS | 91885205 |
| 3 | Cholinesulfuric acid. | C_5_H_13_NO_4_S | 0.93 | 184.0636 | POS | 485 |
| 4 | 3-O-Caffeoylquinic acid | C_16_H_18_O_9_ | 5.75 | 353.0874 | NEG | 1794427 |
| 5 | Vanillic acid | C_8_H_8_O_4_ | 7.24 | 167.0341 | NEG | 8468 |
| 6 | 4-Hydroxybenzoic acid | C_7_H_6_O_3_ | 6.70 | 137.0233 | NEG | 135 |
| 7 | Bisdemethoxycurcumin | C_19_H_16_O_4_ | 12.06 | 309.1114 | POS | 5315472 |
| 8 | _7_beta-Hydroxyrutaecarpine | C_18_H_12_N_3_O_2_ | 12.87 | 303.1010 | POS | 15225951 |
| 9 | Eleutherazine B | C_22_H_36_N_4_O_6_ | 7.22 | 453.2698 | POS | 20839739 |
| 10 | Ginsenoside Rg_1_ | C_42_H_72_O_14_ | 10.54 | 823.4803 | POS | 441923 |
| 11 | 5-Methoxypiperonal | C_9_H_8_O_4_ | 13.35 | 181.0494 | POS | 22016 |
| 12 | ADENOSINE | C_10_H_13_N_5_O_4_ | 4.06 | 268.1035 | POS | 60961 |
| 13 | Carnitine | C_7_H_15_NO_3_ | 0.94 | 162.1123 | POS | 288 |
| 14 | 2,16-Kauranediol 2-O-beta-D-allopyranoside | C_26_H_44_O_7_ | 12.43 | 491.2970 | POS | 73554066 |
| 15 | Vanillin | C_8_H_8_O_3_ | 7.76 | 151.0390 | NEG | 1183 |
| 16 | Scopoletin | C_10_H_8_O_4_ | 8.09 | 237.0400 | NEG_116 | 5280460 |
| 17 | 1-(3,4-Dihydroxyphenyl)-7-(4-hydroxyphenyl)-4-hept | C_19_H_20_O_4_ | 11.18 | 295.1322 | POS | 11570978 |
| 18 | Trigonelline | C_7_H_7_NO_2_ | 1.01 | 138.0549 | POS | 5570 |
| 19 | D-mannitol | C_6_H_14_O_6_ | 0.93 | 181.0709 | NEG | 6251 |
| 20 | Hydroxyanigorufone | C_19_H_12_O_3_ | 11.91 | 287.0710 | NEG | 11471752 |
| 21 | myo-Inositol | C_6_H_12_O_6_ | 1.06 | 179.0552 | NEG | 892 |
| 22 | 4'-Hydroxy-2-O-methylanigorufone | C_20_H_14_O_3_ | 12.10 | 301.0866 | NEG | 71358480 |
| 23 | Magnaldehyde B | C_18_H_16_O_3_ | 12.29 | 245.0957 | POS | 5320888 |
| 24 | L(+)-Ascorbic acid | C_6_H_8_O_6_ | 1.23 | 221.0297 | NEG | 54670067 |
| 25 | Mercaptobenzothiazole | C_7_H_5_NS_2_ | 9.90 | 167.9935 | POS | 697993 |
| 26 | 3,4-Dihydroxyphenylacetic acid | C_8_H_8_O_4_ | 5.09 | 167.0341 | NEG | 547 |
| 27 | D-Glutamic acid | C_5_H_9_NO_4_ | 0.93 | 148.0603 | POS | 23327 |
| 28 | Suavioside A | C_26_H_44_O_8_ | 11.54 | 507.2922 | POS | 73821014 |
| 29 | Sugeroside | C_26_H_42_O_8_ | 11.74 | 505.2764 | POS | 3082543 |
| 30 | 4-Hydroxyphenylpyruvic acid | C_9_H_8_O_4_ | 5.85 | 163.0389 | POS | 979 |
| 31 | 3-Hexen-1-ol O-b-D-glucopyranoside | C_12_H_22_O_6_ | 9.25 | 261.1340 | NEG | 5318045 |
| 32 | 4-Hydroxycinnamamide | C_9_H_9_NO_2_ | 6.18 | 198.0320 | NEG | 16637983 |
| 33 | Xylitol | C_5_H_12_O_5_ | 0.94 | 151.0601 | NEG | 6912 |
| 34 | URIDINE | C_9_H_12_N_2_O_6_ | 2.79 | 243.0618 | NEG | 6029 |
| 35 | D-LEUCINE | C_6_H_13_NO_2_ | 2.01 | 132.1019 | POS | 439524 |
| 36 | linoleic acid | C_18_H_32_O_2_ | 14.89 | 313.2729 | POS | 5280450 |
| 37 | D-Arabinose | C_5_H_10_O_5_ | 0.95 | 133.0495 | POS | 854 |
| 38 | Opuntiol | C_7_H_8_O_4_ | 1.06 | 174.0760 | POS | 10034839 |
| 39 | Dehydroacerogenin C | C_19_H_18_O_3_ | 11.52 | 259.1113 | POS | 154790969 |
| 40 | D-Glucosamine | C_6_H_13_NO_5_ | 0.96 | 180.0864 | POS | 439213 |
| 41 | caffeic acid | C_9_H_8_O_4_ | 6.80 | 163.0389 | POS | 689043 |
| 42 | Lucidone | C_15_H_12_O_4_ | 9.51 | 301.0714 | NEG | 11253859 |
| 43 | D-proline | C_5_H_9_NO_2_ | 1.03 | 116.0708 | POS | 8988 |
| 44 | Guanosine | C_10_H_13_N_5_O_5_ | 2.05 | 284.0983 | POS | 135398635 |
| 45 | Hexylitaconic acid | C_11_H_18_O_4_ | 11.67 | 213.1126 | NEG | 11447214 |
| 46 | 13-Hydroxygermacrone | C_15_H_22_O_2_ | 12.96 | 235.1689 | POS | 10399140 |
| 47 | Aconine | C_25_H_41_NO_9_ | 12.44 | 517.3130 | POS | 20054813 |
| 48 | Danshenol C | C_21_H_20_O_4_ | 11.52 | 301.1216 | POS | 11688609 |
| 49 | 7,8-Dihydroxycoumarin | C_9_H_6_O_4_ | 7.13 | 223.0243 | NEG | 5280569 |
| 50 | 2-Hydroxypalmitic acid | C_16_H_32_O_3_ | 14.74 | 271.2275 | NEG | 92836 |
| 51 | Picrotoxinin | C_15_H_16_O_6_ | 9.54 | 273.0766 | NEG | 442292 |
| 52 | D-Valine | C_5_H_11_NO_2_ | 0.94 | 235.1648 | POS | 71563 |
| 53 | Notoginsenoside R_1_ | C_47_H_80_O_18_ | 10.22 | 955.5221 | POS | 131752529 |
| 54 | Giffonin R | C_19_H_16_O_3_ | 10.74 | 257.0956 | POS | 134715258 |
| 55 | Quinic acid | C_7_H_12_O_6_ | 3.84 | 173.0446 | NEG | 6508 |
| 56 | 4-Epialyxialactone | C_10_H_16_O_4_ | 10.08 | 199.0969 | NEG | 14194344 |
| 57 | Gallic acid | C_7_H_6_O_5_ | 3.11 | 169.0133 | NEG | 370 |
| 58 | Demethoxycurcumin | C_20_H_18_O_5_ | 12.09 | 339.1220 | POS | 5469424 |
| 59 | Stemonidine | C_19_H_29_NO_5_ | 13.29 | 332.1863 | NEG | 24721470 |
| 60 | 1,6-anhydro-b-D-Glucose | C_6_H_10_O_5_ | 1.23 | 207.0503 | NEG | 2724705 |
| 61 | 5,7-Dihydroxyphthalide | C_8_H_6_O_4_ | 6.15 | 211.0242 | NEG | 11062751 |
| 62 | 1-Phenyl-2-propanol | C_9_H_12_O | 12.38 | 119.0857 | POS | 94185 |
| 63 | Dihydrosesamin | C_20_H_20_O_6_ | 9.30 | 355.1183 | NEG | 10871980 |
| 64 | Isoderrone | C_20_H_16_O_5_ | 13.26 | 317.0813 | NEG | 14237660 |
| 65 | 7,8-Benzoflavone | C_19_H_12_O_2_ | 10.78 | 317.0815 | NEG | 11790 |
| 66 | Syringolin A | C_24_H_39_N_5_O_6_ | 12.18 | 535.3238 | POS | 42601513 |
| 67 | Gamabufotalin | C_24_H_34_O_5_ | 12.24 | 403.2450 | POS | 259803 |
| 68 | D-Glucosaminic acid | C_6_H_13_NO_6_ | 0.98 | 160.0603 | POS | 73563 |
| 69 | 7-Deoxyechinosporin | C_10_H_9_NO_4_ | 6.96 | 190.0497 | POS | 11745823 |
| 70 | Methyl gallate | C_8_H_8_O_5_ | 6.19 | 165.0184 | NEG | 7428 |
| 71 | Perilloxin | C_16_H_18_O_4_ | 12.02 | 313.0828 | POS | 10468570 |
| 72 | cinnamaldehyde | C_9_H_8_O | 11.00 | 133.0648 | POS | 637511 |
| 73 | Paeonilactone A | C_10_H_14_O_4_ | 6.75 | 216.1228 | POS | 10081437 |
| 74 | Longicaulenone | C_12_H_18_O_4_ | 7.91 | 249.1108 | POS | 25750965 |
| 75 | p-Hydroxy-5,6-dehydrokawain | C_14_H_12_O_4_ | 10.29 | 225.0554 | NEG | 10243535 |
| 76 | [(3,8,12-trihydroxy-24-oxocholan-24-yl)amino]acetate | C_26_H_42_NO_6_^-^ | 10.54 | 509.2716 | POS | 11834768 |
| 77 | Methyl 4-hydroxy-3-methoxycinnamate | C_11_H_12_O_4_ | 8.67 | 209.0807 | POS | 5357283 |
| 78 | Sarracenin | C_11_H_14_O_5_ | 7.48 | 227.0908 | POS | - |
| 79 | Preisocalamendiol | C_15_H_24_O | 10.54 | 203.1793 | POS | 12305706 |
| 80 | Adenine | C_5_H_5_N_5_ | 1.30 | 136.0618 | POS | 190 |
| 81 | Virginiaebutanolide C | C_11_H_20_O_4_ | 11.23 | 215.1283 | NEG | 40565489 |
| 82 | Mandelic acid | C_8_H_8_O_3_ | 9.70 | 151.0390 | NEG | 1292 |
| 83 | linolenic acid | C_18_H_30_O_2_ | 13.91 | 311.2573 | POS | 5280934 |
| 84 | Lonfuranacid A | C_12_H_20_O_5_ | 9.90 | 243.1233 | NEG | 146116229 |
| 85 | 4,5-Dihydroblumenol A | C_13_H_22_O_3_ | 10.24 | 191.1428 | POS | 21630916 |
| 86 | Pierisformoside B | C_26_H_42_O_8_ | 11.09 | 505.2763 | POS | 155978780 |
| 87 | Glepidotin B | C_20_H_20_O_5_ | 11.44 | 321.1127 | NEG | - |
| 88 | Uracil | C_4_H_4_N_2_O_2_ | 2.87 | 113.0349 | POS | 1174 |
| 89 | ferulic acid | C_10_H_10_O_4_ | 9.59 | 193.0499 | NEG | 445858 |
| 90 | Daphnetin | C_9_H_6_O_4_ | 7.56 | 177.0185 | NEG | 5280569 |
| 91 | 8,9-Didehydro-7-hydroxydolichodial | C_10_H_12_O_3_ | 13.17 | 163.0752 | POS | 3062370 |
| 92 | 2,6,6-Trimethyl-2,4-cycloheptadien-1-one | C_10_H_14_O | 9.30 | 183.1378 | POS | 136330 |
| 93 | Pinocembrin diacetate | C_19_H_16_O_6_ | 9.42 | 385.0925 | NEG | 6546286 |
| 94 | Przewaquinone C | C_18_H_16_O_4_ | 12.18 | 261.0905 | POS | 126071 |
| 95 | Cyclocerberidol | C_9_H_16_O_4_ | 7.17 | 153.0909 | POS | 14466834 |
| 96 | N-Isobutyl-2,4,12-octadecatrienamide | C_22_H_39_NO | 12.91 | 378.2750 | POS | 25221579 |
| 97 | 3'-Hydroxyxanthyletin | C_14_H_12_O_4_ | 10.30 | 227.0699 | POS | 129069558 |
| 98 | trans-5-Hydroxyferulic acid | C_10_H_10_O_5_ | 7.79 | 209.0449 | NEG | 446834 |
| 99 | Methyl hexadecanoate | C_17_H_34_O_2_ | 13.20 | 315.2535 | NEG | 8181 |
| 100 | Excavatin M | C_19_H_20_O_7_ | 7.81 | 359.1133 | NEG | 15871351 |
| 101 | hypoxanthine | C_5_H_4_N_4_O | 4.26 | 137.0458 | POS | 135398638 |
| 102 | Syringic acid | C_9_H_10_O_5_ | 7.50 | 197.0448 | NEG | 10742 |
| 103 | Ethyl vanillin acetate | C_11_H_12_O_4_ | 8.68 | 207.0656 | NEG | 155708 |
| 104 | ent-14,15-Dinor-13-oxolabda-8(17),11-dien-18-oic acid | C_18_H_26_O_3_ | 10.27 | 308.2213 | POS | 91885074 |
| 105 | ETHYL CAFFEATE | C_11_H_12_O_4_ | 10.13 | 207.0656 | NEG | 5317238 |
| 106 | 2-C-Methyl-D-erythrono-1,4-lactone | C_5_H_8_O_4_ | 1.26 | 133.0496 | POS | 11126294 |
| 107 | 4-AMINOBUTYRIC ACID | C_4_H_9_NO_2_ | 0.94 | 104.0710 | POS | 119 |
| 108 | N-a-Acetyl-L-arginine | C_8_H_16_N_4_O_3_ | 1.30 | 280.1385 | POS | 67427 |
| 109 | Thymolglucoside | C_16_H_24_O_6_ | 11.30 | 351.1217 | POS | 88687 |
| 110 | 7-Methoxycoumarin | C_10_H_8_O_3_ | 10.11 | 221.0450 | NEG | 10748 |
| 111 | 7-Aminocephalosporanic acid | C_10_H_12_N_2_O_5_S | 15.06 | 305.0800 | POS | - |
| 112 | Citropten | C_11_H_10_O_4_ | 8.25 | 251.0557 | NEG | 2775 |
| 113 | Clostebol acetate | C_21_H_29_ClO_3_ | 1.08 | 401.1294 | NEG | 13327 |
| 114 | L-glutamic acid | C_5_H_9_NO_4_ | 1.35 | 148.0603 | POS | 33032 |
| 115 | Ginsenoside Re | C_48_H_82_O_1_8 | 10.45 | 969.5373 | POS | - |
| 116 | Puerol A | C_17_H_14_O_5_ | 10.41 | 279.0659 | NEG | 14691941 |
| 117 | Homononactinic acid | C_11_H_20_O_4_ | 9.77 | 181.1222 | POS | 10889304 |
| 118 | Gymnestrogenin | C_30_H_50_O_5_ | 13.81 | 513.3543 | POS | 15560302 |
| 119 | 3-Hydroxy-p-menth-1-en-6-one | C_10_H_16_O_2_ | 11.68 | 151.1117 | POS | 14106048 |
| 120 | Resedine | C_9_H_9_NO_2_ | 8.62 | 146.0600 | POS | 202193 |
| 121 | Inosine | C_10_H_12_N_4_O_5_ | 2.55 | 267.0731 | NEG | 135398641 |
| 122 | Acetylglycine | C_4_H_7_NO_3_ | 0.89 | 159.0763 | POS | 10972 |
| 123 | Microminutin | C_15_H_12_O_5_ | 8.91 | 317.0662 | NEG | 5319827 |
| 124 | Cucumegastigmane I | C_13_H_20_O_4_ | 10.01 | 241.1431 | POS | 16105430 |
| 125 | Amentoflavone | C_30_H_18_O_10_ | 0.98 | 539.0998 | POS | 5281600 |
| 126 | Alternariol | C_14_H_10_O_5_ | 8.69 | 241.0492 | POS | 5359485 |
| 127 | Shikimic Acid | C_7_H_10_O_5_ | 6.85 | 173.0447 | NEG | 8742 |
| 128 | Clausine I | C_14_H_10_NO_3_ | 14.05 | 239.0591 | NEG | - |
| 129 | 2-O-Methylanigorufone | C_20_H_14_O_2_ | 11.52 | 269.0956 | POS | - |
| 130 | 2-Adamantanone | C_10_H_14_O | 8.79 | 195.1019 | NEG | 64151 |
| 131 | Protocatechualdehyde | C_7_H_6_O_3_ | 6.33 | 139.0389 | POS | 8768 |
| 132 | (E)-Cinnamic acid | C_9_H_8_O_2_ | 8.51 | 147.0441 | NEG | 444539 |
| 133 | artemisinin | C_15_H_22_O_5_ | 8.93 | 300.1799 | POS | 68827 |
| 134 | 3-Hydroxyperillaldehyde | C_10_H_14_O_2_ | 8.35 | 149.0961 | POS | 85247137 |
| 135 | 4-Oxododecanedioic acid | C_12_H_20_O_5_ | 10.02 | 225.1127 | NEG | 13213508 |
| 136 | Tinospin E | C_20_H_20_O_6_ | 11.70 | 321.1113 | POS | 71473355 |
| 137 | Deacetylorientalide | C_19_H_22_O_7_ | 10.81 | 401.0988 | POS | 23815409 |
| 138 | Coniferaldehyde | C_10_H_10_O_3_ | 8.68 | 179.0702 | POS | 5280536 |
| 139 | Cleroindicin B | C_8_H_14_O_3_ | 6.68 | 141.0910 | POS | 184824 |
| 140 | p-Hydroxybenzaldehyde | C_7_H_6_O_2_ | 7.29 | 123.0443 | POS | 126 |
| 141 | Bergapten | C_12_H_8_O_4_ | 10.44 | 261.0402 | NEG | 2355 |
| 142 | Artemisinic acid | C_15_H_22_O_2_ | 12.17 | 235.1690 | POS | 10922465 |
| 143 | 5-Hydroxy-2-methylchromone | C_10_H_8_O_3_ | 7.75 | 177.0545 | POS | 821390 |
| 144 | Ursolic acid | C_30_H_48_O_3_ | 14.74 | 457.3666 | POS | - |
| 145 | 5-Acetylsalicylic acid | C_9_H_8_O_4_ | 6.69 | 181.0494 | POS | 83151 |
| 146 | 7-Methoxy-1-naphthaleneacetic acid | C_13_H_12_O_3_ | 8.29 | 197.0601 | NEG | 6862 |
| 147 | Enalin A | C_10_H_10_O_4_ | 6.81 | 177.0545 | POS | 12084864 |
| 148 | 4-Hydroxycinnamic acid | C_9_H_8_O_3_ | 6.45 | 163.0391 | NEG | 637542 |
| 149 | trans-3-Indoleacrylic acid | C_11_H_8_NO_2_ | 8.68 | 207.0293 | NEG | 5375048 |
| 150 | D-altrofurano-heptulose-3 | C_7_H_14_O_7_ | 0.95 | 245.0428 | NEG | 145865405 |
| 151 | Alisol F | C_30_H_48_O_5_ | 13.48 | 511.3391 | POS | - |
| 152 | Esculetin | C_9_H_6_O_4_ | 7.55 | 179.0337 | POS | 5281416 |
| 153 | Justicidin A | C_22_H_18_O_7_ | 11.29 | 375.0870 | NEG | - |
| 154 | 16-[(aminocarbonyl)hydrazono]stachan-18-oic acid | C_21_H_33_N_3_O_3_ | 14.54 | 376.2595 | POS | - |
| 155 | 3-Hydroxymethylenetanshinquinone | C_18_H_14_O_4_ | 11.47 | 339.0869 | NEG | 5318290 |
| 156 | piperic acid | C_12_H_10_O_4_ | 8.16 | 199.0393 | NEG | 5370536 |
| 157 | Calystegine B_2_ | C_7_H_13_NO_4_ | 1.24 | 140.0706 | POS | 124434 |
| 158 | Ethyl alpha-D-ribo-hex-3-ulopyranoside | C_8_H_14_O_6_ | 6.82 | 171.0651 | POS | - |
| 159 | P-Anisic acid | C_8_H_8_O_3_ | 7.56 | 151.0390 | NEG | - |
| 160 | Glycolaldehyde dimer | C_4_H_8_O_4_ | 1.21 | 103.0394 | POS | 186078 |
| 161 | Isosclerone | C_10_H_10_O_3_ | 7.23 | 196.0967 | POS | 13369486 |
| 162 | 1-Hydroxypyrene | C_16_H_10_O | 12.37 | 263.0710 | NEG | - |
| 163 | 1,6-Dioxaspiro[4.5]decan-2-methanol | C_9_H_16_O_3_ | 8.65 | 217.1075 | NEG | 588029 |
| 164 | (_2_RS)-Lotaustralin | C_11_H_19_NO_6_ | 3.77 | 294.1540 | POS | 441467 |
| 165 | 20(S),24(R)-Ocotillol | C_30_H_52_O_5_ | 14.32 | 515.3702 | POS | 15886258 |
| 166 | 1-(4-Hydroxy-2,2-dimethylchroman-6-yl)ethanone | C_13_H_16_O_3_ | 12.72 | 221.1170 | POS | 129887823 |
| 167 | trans-2-Tridecene-1,13-dioic acid | C_13_H_22_O_4_ | 11.98 | 241.1440 | NEG | 23091819 |
| 168 | Kigelinone | C_19_H_16_O_4_ | 11.29 | 289.0866 | NEG | 442752 |
| 169 | Armillarisin A | C_12_H_10_O_5_ | 6.70 | 279.0506 | NEG | 5320192 |
| 170 | Bergaptol | C_11_H_6_O_4_ | 9.04 | 247.0244 | NEG | 5280371 |
| 171 | Asarylaldehyde | C_10_H_12_O_4_ | 7.28 | 197.0807 | POS | 20525 |
| 172 | Ethyl alpha-D-apiofuranoside | C_7_H_14_O_5_ | 1.37 | 161.0807 | POS | - |
| 173 | 11-Hydroxyjasmonic acid | C_12_H_18_O_4_ | 9.25 | 259.1536 | POS | 15127090 |
| 174 | D-Aspartic acid | C_4_H_7_NO_4_ | 0.90 | 134.0448 | POS | 83887 |
| 175 | Axillaridine | C_18_H_27_NO_6_ | 13.60 | 371.2171 | POS | 179398 |
| 176 | (+)-Mellein | C_10_H_10_O_3_ | 8.68 | 177.0548 | NEG | 28516 |
| 177 | Senkyunolide I | C_12_H_16_O_4_ | 7.77 | 223.0970 | NEG | 11521428 |
| 178 | Sanggenone H | C_20_H_18_O_6_ | 12.11 | 319.0958 | POS | 90681446 |
| 179 | Isopsoralen | C_11_H_6_O_3_ | 8.62 | 187.0388 | POS | 10658 |
| 180 | Furanofukinin | C_16_H_24_O_2_ | 12.19 | 293.1755 | NEG | 78385403 |
| 181 | 5-Methyl-7-methoxyisoflavone | C_17_H_14_O_3_ | 11.22 | 231.0801 | POS | 2734290 |
| 182 | Wilforlide A | C_30_H_46_O_3_ | 14.66 | 455.3495 | POS | - |
| 183 | Praeruptorin A | C_21_H_22_O_7_ | 9.01 | 409.1248 | POS | 38347607 |
| 184 | m-Digallic acid | C_14_H_10_O_9_ | 9.92 | 303.0143 | NEG | - |
| 185 | L-allo-Threonine | C_4_H_9_NO_3_ | 0.90 | 120.0657 | POS | 99289 |
| 186 | N-Acetylleucine | C_8_H_15_NO_3_ | 7.92 | 174.1124 | POS | 70912 |
| 187 | 1-Linoleoyl-sn-glycero-3-phosphorylcholine | C_26_H_50_NO_7_P | 14.19 | 520.3389 | POS | 11005824 |
| 188 | Taiwapyrone | C_10_H_14_O_4_ | 8.95 | 163.0753 | POS | 101316864 |
| 189 | Phenylacetylglycine | C_10_H_11_NO_3_ | 8.11 | 174.0552 | NEG | 68144 |
| 190 | Teuvincenone H | C_20_H_20_O_6_ | 10.55 | 337.1078 | NEG | 50899164 |
| 191 | Massarilactone B | C_11_H_14_O_5_ | 7.70 | 207.0656 | NEG | 10105097 |
| 192 | (S)-Leucic acid | C_6_H_12_O_3_ | 0.87 | 150.1124 | POS | - |
| 193 | Syringaldehyde | C_9_H_10_O_4_ | 7.94 | 181.0498 | NEG | 8655 |
| 194 | Retusin | C_19_H_18_O_7_ | 9.29 | 341.1010 | POS | 5352005 |
| 195 | Dihydrocoriandrin | C_13_H_12_O_4_ | 8.64 | 213.0551 | NEG | 14134311 |
| 196 | 3-O-Methylanhydrotuberosin | C_21_H_18_O_4_ | 13.03 | 379.0911 | POS | - |
| 197 | Irisflorentin | C_20_H_18_O_8_ | 8.31 | 369.0958 | POS | 170569 |
| 198 | 2-Pentadecenedioic acid | C_15_H_26_O_4_ | 12.52 | 251.1649 | NEG | 12867460 |
| 199 | 1-Oxo-4-hydroxy-2-en-4-ethylcyclohexa-5,8-olide | C_8_H_8_O_4_ | 2.82 | 186.0759 | POS | 85844078 |
| 200 | Magnolioside | C_16_H_18_O_9_ | 8.93 | 319.0805 | POS | 3084335 |
| 201 | Narchinol B | C_12_H_16_O_3_ | 8.77 | 191.1064 | POS | 56835095 |
| 202 | Atractylenolide II | C_15_H_20_O_2_ | 13.59 | 277.1441 | NEG | 14448070 |
| 203 | 6-O-Ethyltetradymodiol | C_17_H_26_O_3_ | 11.82 | 323.1854 | NEG | 162984443 |
| 204 | Dehydrologanin | C_17_H_24_O_10_ | 10.25 | 423.1082 | NEG | 11968402 |
| 205 | Desmethylbellidifolin | C_13_H_8_O_6_ | 10.16 | 259.0245 | NEG | 5281626 |
| 206 | Erythrinin G | C_20_H_18_O_6_ | 11.57 | 335.0921 | NEG | 133561644 |
| 207 | Noroxyhydrastinine | C_10_H_9_NO_3_ | 7.58 | 236.0560 | NEG | 89047 |
| 208 | Buspirone free base | C_21_H_31_N_5_O_2_ | 13.03 | 427.2809 | POS | 2477 |
| 209 | Ethyl 3,4-dihydroxybenzoate | C_9_H_10_O_4_ | 9.20 | 181.0498 | NEG | 77547 |
| 210 | ethyl 2-(1-hydroxy-4-oxocyclohexa-2,5-dien-1-yl)acetate | C_10_H_12_O_4_ | 7.24 | 195.0654 | NEG | 100323 |
| 211 | meso-dihydroguaiaretic acid | C_20_H_26_O_4_ | 12.51 | 353.1692 | POS | 476856 |
| 212 | Rengynic acid | C_8_H_14_O_4_ | 7.78 | 139.0753 | POS | 54033324 |
| 213 | 6-Shogaol | C_17_H_24_O_3_ | 12.58 | 259.1667 | POS | 5281794 |
| 214 | 6-Demethoxycleomiscosin A | C_19_H_16_O_7_ | 9.24 | 401.0873 | NEG | - |
| 215 | Kirenol | C_20_H_34_O_4_ | 13.65 | 339.2499 | POS | 15736732 |
| 216 | Evodol | C_26_H_28_O_9_ | 14.21 | 529.1425 | POS | - |
| 217 | D-Serine | C_3_H_7_NO_3_ | 0.90 | 147.0763 | POS | 71077 |
| 218 | Protoescigenin | C_30_H_50_O_6_ | 13.47 | 529.3495 | POS | 15560300 |
| 219 | Sterebin A | C_18_H_30_O_4_ | 11.81 | 309.2067 | NEG | 21681091 |
| 220 | Cryptomoscatone D_2_ | C_17_H_20_O_4_ | 13.09 | 327.0984 | POS | 76524569 |
| 221 | Isovanillic acid | C_8_H_8_O_4_ | 4.39 | 213.0398 | NEG | 12575 |
| 222 | 8-Methoxybonducellin | C_18_H_16_O_5_ | 8.90 | 357.0977 | NEG | 73353608 |
| 223 | 13,14,15,16-Tetranor-8(17)-labden-12-oic acid | C_16_H_26_O_2_ | 12.53 | 295.1909 | NEG | 14380023 |
| 224 | 3-Chloro-1-(4-octylphenyl)-propanone | C_17_H_25_ClO | 13.97 | 263.1571 | POS | 70700719 |
| 225 | Isopentyl b-D-glucoside | C_11_H_22_O_6_ | 10.08 | 215.1276 | POS | 10848285 |
| 226 | 9-Hydroxy-a-lapachone | C_15_H_14_O_4_ | 7.74 | 303.0871 | NEG | - |
| 227 | Isofraxoside | C_16_H_18_O_10_ | 11.23 | 393.0811 | POS | 11508953 |
| 228 | 5-Dehydroxyparatocarpin K | C_20_H_18_O_4_ | 12.53 | 303.1023 | NEG | 11515298 |
| 229 | VERATRIC ACID | C_9_H_10_O_4_ | 8.55 | 181.0498 | NEG | 7121 |
| 230 | Citrusinol | C_20_H_16_O_6_ | 10.36 | 317.0801 | POS | 44259051 |
| 231 | falcarindiol | C_17_H_24_O_2_ | 12.59 | 261.1827 | POS | 5281148 |
| 232 | Benzoylphenylalanine | C_16_H_15_NO_3_ | 10.55 | 268.0976 | NEG | 97370 |
| 233 | 4-(cis)-Acetyl-3,6,8-trihydroxy-3-methyldihydronaphthalenone | C_13_H_14_O_5_ | 9.14 | 231.0658 | NEG | 10777052 |
| 234 | L-TYROSINE | C_9_H_11_NO_3_ | 2.17 | 180.0657 | NEG | 6057 |
| 235 | N-Acetyl-phenylalanine | C_11_H_13_NO_3_ | 8.27 | 206.0816 | NEG | 74839 |
| 236 | docosapentaenoic acid | C_22_H_34_O_2_ | 13.62 | 375.2278 | POS | 5497182 |
| 237 | Eupatoriochromene | C_13_H_14_O_3_ | 9.03 | 219.1009 | POS | 100768 |
| 238 | Ruscogenin | C_27_H_42_O_4_ | 13.52 | 453.2972 | POS | 441893 |
| 239 | 2-Oxo-3-phenylpropanoic acid | C_9_H_8_O_3_ | 6.51 | 147.0439 | POS | - |
| 240 | Dianthoside | C_12_H_16_O_8_ | 1.21 | 306.1177 | POS | 5316639 |
| 241 | Santin | C_18_H_16_O_7_ | 12.18 | 345.0978 | POS | 5281695 |
| 242 | Cordycepin | C_10_H_13_N_5_O_3_ | 4.20 | 252.1086 | POS | 6303 |
| 243 | Erythbidin A | C_20_H_20_O_4_ | 11.69 | 323.1284 | NEG | 15391906 |
| 244 | Clausenin | C_14_H_12_O_5_ | 9.98 | 241.0502 | NEG | 5315948 |
| 245 | Saropyrone | C_16_H_16_O_5_ | 8.87 | 287.0921 | NEG | - |
| 246 | 2-Hydroxy Hippuric Acid | C_9_H_9_NO_4_ | 4.37 | 213.0867 | POS | 10253 |
| 247 | 7,8-Dihydrokawain-5-ol | C_14_H_16_O_4_ | 12.27 | 287.0678 | POS | 10944777 |
| 248 | Panasenoside | C_27_H_30_O_1_6 | 7.24 | 611.1599 | POS | 9986191 |
| 249 | Flavin mononucleotide | C_17_H_19_N_4_O_6_ | 13.13 | 393.1656 | POS | 643976 |
| 250 | (+)-catechin | C_15_H_14_O_6_ | 6.45 | 289.0715 | NEG | 9064 |
| 251 | Phyllanthurinolactone | C_14_H_18_O_8_ | 8.07 | 353.0645 | POS | 10957981 |
| 252 | 5-O-Cinnamoylquinic acid | C_16_H_18_O_7_ | 8.97 | 345.0961 | POS | 162642276 |
| 253 | Cearoin | C_14_H_12_O_4_ | 11.04 | 225.0555 | NEG | 3938139 |
| 254 | Kaempferol 3,4',7-triacetate | C_21_H_16_O_9_ | 8.84 | 445.1110 | POS | - |
| 255 | 5-deoxy Thymidine | C_10_H_14_N_2_O_4_ | 5.53 | 209.0918 | POS | 65120 |
| 256 | 2-Methoxystypandrone | C_14_H_12_O_5_ | 8.69 | 259.0609 | NEG | 158739 |
| 257 | Damascenone | C_13_H_18_O | 10.51 | 173.1325 | POS | 5366074 |
| 258 | Radicicol | C_18_H_17_ClO_6_ | 11.30 | 385.0468 | NEG | - |
| 259 | Verminoside | C_24_H_28_O_13_ | 8.26 | 569.1252 | POS | 12000883 |
| 260 | p-Coumaric Acid Ethyl Ester | C_11_H_12_O_3_ | 10.86 | 191.0705 | NEG | - |
| 261 | 4 | C_20_H_14_O_5_ | 12.23 | 333.0764 | NEG | - |
| 262 | xanthosine | C_10_H_12_N_4_O_6_ | 4.20 | 283.0680 | NEG | 64959 |
| 263 | (-)-Epicatechin | C_15_H_14_O_6_ | 6.45 | 291.0857 | POS | 72276 |
| 264 | Ochratoxin B | C_20_H_19_NO_6_ | 6.93 | 370.1273 | POS | 20966 |
| 265 | Acetyl-11-keto-Beta-boswellic acid | C_32_H_48_O_5_ | 12.60 | 513.3542 | POS | 9847548 |
| 266 | norepinephrine | C_8_H_11_NO_3_ | 4.58 | 214.0715 | NEG | 439260 |
| 267 | Laetisaric acid | C_18_H_32_O_3_ | 14.82 | 261.2206 | POS | 5281117 |
| 268 | Furomollugin | C_14_H_10_O_4_ | 9.03 | 287.0557 | NEG | - |
| 269 | (E)-Cinnamyl Acetate | C_11_H_12_O_2_ | 12.29 | 194.1174 | POS | - |
| 270 | prim-O-Glucosylangelicain | C_21_H_26_O_11_ | 8.88 | 499.1201 | POS | 91895378 |
| 271 | Chrysin 6-C-arabinoside 8-C-glucoside | C_26_H_28_O_1_3 | 9.47 | 549.1593 | POS | 21722007 |
| 272 | Eucamalol | C_10_H_16_O_2_ | 10.30 | 213.1126 | NEG | 12426239 |
| 273 | Anigorufone | C_19_H_12_O_2_ | 9.87 | 273.0904 | POS | - |
| 274 | gamma-Linolenic acid | C_18_H_30_O_2_ | 13.81 | 323.2221 | NEG | 5280933 |
| 275 | Isoboonein | C_9_H_14_O_3_ | 5.58 | 215.0919 | NEG | 10899112 |
| 276 | Isoanhydroicaritin | C_21_H_20_O_6_ | 9.96 | 369.1313 | POS | 5322079 |
| 277 | Pterodondiol | C_15_H_28_O_2_ | 13.50 | 285.2067 | NEG | 10879263 |
| 278 | 3,4-Dihydroxy-2-O-methylanigorufone | C_20_H_14_O_4_ | 13.07 | 301.0852 | POS | 125115445 |
| 279 | Cryptochlorogenic acid | C_16_H_18_O_9_ | 7.34 | 335.0769 | NEG | 9798666 |
| 280 | Nardosinone | C_15_H_22_O_3_ | 10.25 | 273.1458 | POS | 168136 |
| 281 | Gardoside | C_16_H_22_O_10_ | 10.03 | 339.1067 | POS | 46173850 |
| 282 | 1,2-Dihydrotanshinquinone | C_18_H_14_O_3_ | 11.14 | 243.0800 | POS | - |
| 283 | Mearnsetin | C_16_H_12_O_8_ | 10.09 | 313.0351 | NEG | 10359384 |
| 284 | Psoromic acid | C_18_H_14_O_8_ | 7.54 | 323.0542 | POS | 23725 |
| 285 | L-Pyroglutamic acid | C_5_H_7_NO_3_ | 1.45 | 257.0774 | NEG | 7405 |
| 286 | Hydrourushiol | C_21_H_36_O_2_ | 13.13 | 365.2689 | NEG | 68118 |
| 287 | Aurantiamide | C_25_H_26_N_2_O_3_ | 12.30 | 383.1760 | NEG | 185904 |
| 288 | Obscuraminol E | C_16_H_33_NO | 14.85 | 288.2890 | POS | 134715064 |
| 289 | Hamaudol | C_15_H_16_O_5_ | 11.48 | 241.0856 | POS | 164722 |
| 290 | Nordalbergin | C_15_H_10_O_4_ | 9.49 | 255.0648 | POS | 5320203 |
| 291 | Guanine | C_5_H_5_N_5_O | 1.88 | 150.0411 | NEG | 135398634 |
| 292 | Ethyl brevifolincarboxylate | C_15_H_12_O_8_ | 12.36 | 338.0871 | POS | - |
| 293 | Citrate | C_6_H_8_O_7_ | 3.70 | 210.0611 | POS | 31348 |
| 294 | 2,4-Dihydroxy-6-methoxy-3-formylacetophenone | C_10_H_10_O_5_ | 6.28 | 255.0506 | NEG | 610933 |
| 295 | Oridonin | C_20_H_28_O_6_ | 13.50 | 365.1929 | POS | - |
| 296 | 2-Hydroxy-4-methoxybenzaldehyde | C_8_H_8_O_3_ | 6.72 | 185.0808 | POS | 69600 |
| 297 | N-Acetyl-L-aspartic acid | C_6_H_9_NO_5_ | 1.28 | 158.0447 | POS | 65065 |
| 298 | Kumatakenin | C_17_H_14_O_6_ | 7.89 | 337.0698 | POS | 5318869 |
| 299 | Phellolactone | C_13_H_14_O_8_ | 6.75 | 281.0650 | POS | 102004720 |
| 300 | Traumatic Acid | C_12_H_20_O_4_ | 11.86 | 227.1283 | NEG | 5283028 |
| 301 | Equisetin | C_22_H_31_NO_4_ | 14.33 | 372.2175 | NEG | 54684703 |
| 302 | baicalein | C_15_H_10_O_5_ | 8.80 | 271.0596 | POS | 5281605 |
| 303 | Isopimpinellin | C_13_H_10_O_5_ | 9.36 | 227.0344 | NEG | 68079 |
| 304 | 7-hydroxy-4-methyl-8-nitrocoumarin | C_10_H_7_NO_5_ | 0.84 | 242.0067 | NEG | 5376327 |
| 305 | Sarmentosin | C_11_H_17_NO_7_ | 1.55 | 240.0863 | POS | 5281123 |
| 306 | Paeoniflorigenone | C_17_H_18_O_6_ | 9.90 | 301.1064 | POS | - |
| 307 | Lamiophlomiol A | C_11_H_14_O_6_ | 7.29 | 241.0714 | NEG | 125923 |
| 308 | 1-Oleoyl-sn-glycero-3-phosphocholine | C_26_H_52_NO_7_P | 14.43 | 522.3540 | POS | 16081932 |
| 309 | Curdione | C_15_H_24_O_2_ | 4.57 | 259.1647 | POS | 6441391 |
| 310 | Pedatisectine F | C_9_H_14_N_2_O_4_ | 3.52 | 215.1024 | POS | 12285902 |
| 311 | 1-Methylinosine | C_11_H_14_N_4_O_5_ | 7.84 | 317.0662 | NEG | 65095 |
| 312 | Crotanecine | C_8_H_13_NO_3_ | 1.30 | 213.1231 | POS | 394146 |
| 313 | 2-Methoxynaphthoquinone | C_11_H_8_O_3_ | 8.95 | 233.0452 | NEG | 16871 |
| 314 | Tanshindiol A | C_18_H_16_O_5_ | 9.76 | 293.0814 | NEG | 16730071 |
| 315 | Isoorientin | C_21_H_20_O_11_ | 8.23 | 447.0929 | NEG | 114776 |
| 316 | Scopolin | C_16_H_18_O_9_ | 7.34 | 377.0835 | POS | 439514 |
| 317 | 3-O-Acetylpinobanksin | C_17_H_14_O_6_ | 7.49 | 337.0697 | POS | 148556 |
| 318 | Nortanshinone | C_17_H_12_O_4_ | 10.92 | 325.0713 | NEG | 10062187 |
| 319 | ellagic acid | C_14_H_6_O_8_ | 9.37 | 300.9986 | NEG | 5281855 |
| 320 | 10-Gingerol | C_21_H_34_O_4_ | 12.83 | 373.2341 | POS | 168115 |
| 321 | 2,6-Diaminoheptanedioic acid | C_7_H_14_N_2_O_4_ | 1.01 | 191.1024 | POS | 865 |
| 322 | Mogrol | C_30_H_52_O_4_ | 13.85 | 499.3750 | POS | - |
| 323 | Methyl 2-(2-hydroxyphenyl)acetate | C_9_H_10_O_3_ | 8.08 | 208.0967 | POS | - |
| 324 | ophiopogonone A | C_18_H_14_O_6_ | 10.89 | 349.0698 | POS | 10087732 |
| 325 | Sinapinic acid | C_11_H_12_O_5_ | 9.24 | 447.1292 | NEG | 637775 |
| 326 | 3-O-Ethyl-L-ascorbic acid | C_8_H_12_O_6_ | 5.89 | 185.0448 | NEG | 150736 |
| 327 | 4-Hydroxybenzaldehyde rhamnoside | C_13_H_16_O_6_ | 5.91 | 286.1278 | POS | 11777785 |
| 328 | 2-Prenylhydroquinone-1-glucoside | C_17_H_24_O_7_ | 8.60 | 361.1266 | NEG | - |
| 329 | [12]-Dehydrogingerdione | C_23_H_34_O_4_ | 11.76 | 438.2628 | POS | 154791045 |
| 330 | emodin | C_15_H_10_O_5_ | 13.42 | 269.0453 | NEG | 3220 |
| 331 | Tutin | C_15_H_18_O_6_ | 7.74 | 259.0960 | POS | 75729 |
| 332 | Eupalinilide B | C_20_H_24_O_6_ | 8.74 | 361.1613 | POS | 11245337 |
| 333 | (-)-Gallocatechin | C_15_H_14_O_7_ | 5.20 | 307.0805 | POS | 9882981 |
| 334 | 2-phenyl-_4_H-benzo[h]chromen-4-one | C_19_H_12_O_2_ | 12.56 | 271.0762 | NEG | - |
| 335 | 3,7-Di-O-methylquercetin | C_17_H_14_O_7_ | 8.75 | 311.0556 | NEG | 5280417 |
| 336 | Hydroxygenkwanin | C_16_H_12_O_6_ | 11.15 | 299.0556 | NEG | 5318214 |
| 337 | Koaburaside | C_14_H_20_O_9_ | 11.72 | 367.0816 | NEG | 5318820 |
| 338 | 3-Hydroxy-L-tyrosine | C_9_H_11_NO_4_ | 6.64 | 180.0654 | POS | 6047 |
| 339 | Alpha-Asarone | C_12_H_16_O_3_ | 11.17 | 253.1077 | NEG | 636822 |
| 340 | chrysin | C_15_H_10_O_4_ | 10.95 | 253.0503 | NEG | 5281607 |
| 341 | beta-Jonone | C_13_H_20_O | 13.11 | 237.1490 | NEG | - |
| 342 | Methyl ferulate | C_11_H_12_O_4_ | 8.39 | 253.0714 | NEG | 5357283 |
| 343 | 1,2-Didehydrocryptotanshinone | C_19_H_18_O_3_ | 11.38 | 339.1232 | NEG | - |
| 344 | 3-O-Debenzoylzeylenone | C_14_H_14_O_6_ | 5.35 | 323.0767 | NEG | 122184806 |
| 345 | L-Aspartic Acid 4-Benzyl Ester | C_11_H_13_NO_4_ | 4.87 | 222.0766 | NEG | 101186 |
| 346 | Scillascillin | C_17_H_12_O_6_ | 8.23 | 357.0610 | NEG | 75492722 |
| 347 | Procyanidin B_1_ | C_30_H_26_O_1_2 | 6.54 | 577.1350 | NEG | 11250133 |
| 348 | Monocillinol B | C_11_H_13_NO_5_ | 6.22 | 240.0857 | POS | 10060146 |
| 349 | Robinetin | C_15_H_10_O_7_ | 8.93 | 303.0492 | POS | - |
| 350 | Tanshindiol B | C_18_H_16_O_5_ | 10.63 | 293.0817 | NEG | 5321620 |
| 351 | Methyl asterrate | C_18_H_18_O_8_ | 9.19 | 327.0853 | POS | 5249326 |
| 352 | Daidzein diacetate | C_19_H_14_O_6_ | 12.02 | 319.0606 | NEG | - |
| 353 | Emodin-8-O-beta-gentiobioside | C_27_H_30_O_15_ | 7.60 | 593.1510 | NEG | 71587230 |
| 354 | Glabrene | C_20_H_18_O_4_ | 11.87 | 321.1128 | NEG | 480774 |
| 355 | Fibraurin | C_20_H_20_O_7_ | 12.41 | 407.0918 | NEG | 21626387 |
| 356 | Erigoster B | C_26_H_24_O_13_ | 9.32 | 565.0964 | NEG | - |
| 357 | 3-O-Acetylpadmatin | C_18_H_16_O_8_ | 8.86 | 343.0800 | POS | 10406203 |
| 358 | Jangomolide | C_26_H_28_O_8_ | 9.44 | 489.1552 | NEG | 14240958 |
| 359 | Cynaroside | C_21_H_20_O_11_ | 9.84 | 447.0928 | NEG | 5280637 |
| 360 | Coriandrin | C_13_H_10_O_4_ | 6.94 | 229.0501 | NEG | 119586 |
| 361 | 3-Methyladipic acid | C_7_H_12_O_4_ | 6.34 | 205.0711 | NEG | 12292 |
| 362 | Verbasoside | C_20_H_30_O_12_ | 7.61 | 485.1620 | POS | 11754080 |
| 363 | 17-Hydroxyisolathyrol | C_20_H_30_O_5_ | 11.77 | 349.1990 | NEG | 127256191 |
| 364 | Neposide | C_19_H_22_O_8_ | 8.33 | 359.1135 | NEG | 12313291 |
| 365 | Przewaquinone A | C_19_H_18_O_4_ | 11.13 | 291.1022 | NEG | - |
| 366 | Echioidinin | C_16_H_12_O_5_ | 9.26 | 285.0753 | POS | 15559079 |
| 367 | 1-(4-Hydroxybenzoyl)glucose | C_13_H_16_O_8_ | 4.61 | 345.0822 | NEG | 14132342 |
| 368 | Leucanthogenin | C_17_H_14_O_8_ | 7.44 | 347.0752 | POS | - |
| 369 | Viscidulin I | C_15_H_10_O_7_ | 9.04 | 301.0346 | NEG | 5320471 |
| 370 | 3'-hydroxyPuerarin | C_21_H_20_O_10_ | 8.75 | 433.1122 | POS | 5748205 |
| 371 | 1_1_S,12-Dihydroxyspirovetiv-1(10)-en-2-one | C_15_H_24_O_3_ | 12.63 | 251.1649 | NEG | - |
| 372 | Ethyl gallate | C_9_H_10_O_5_ | 7.98 | 197.0448 | NEG | 13250 |
| 373 | Kaempferol 3-glucoside 7-rhamnoside | C_27_H_30_O_15_ | 7.60 | 595.1649 | POS | 57390614 |
| 374 | Gardenine | C_11_H_13_NO_4_ | 9.56 | 258.0532 | NEG | 197414 |
| 375 | Alpinoid D | C_20_H_20_O_3_ | 11.34 | 353.1391 | NEG | 38363343 |
| 376 | LINAMARIN | C_10_H_17_NO_6_ | 1.30 | 289.1388 | POS | 11128 |
| 377 | Ophiopogonanone A | C_18_H_16_O_6_ | 7.31 | 311.0906 | POS | 9996586 |
| 378 | Europine N-oxide | C_16_H_27_NO_7_ | 8.67 | 346.1865 | POS | - |
| 379 | Bergaptol glucoside | C_17_H_16_O_9_ | 10.54 | 329.0649 | POS | - |
| 380 | Eriodictyol | C_15_H_12_O_6_ | 5.19 | 289.0700 | POS | 440735 |
| 381 | Coixol | C_8_H_7_NO_3_ | 4.41 | 210.0401 | NEG | 10772 |
| 382 | Inulicin | C_17_H_24_O_5_ | 9.51 | 350.1963 | POS | 75528891 |
| 383 | 8-O-Acetyltorilolone | C_17_H_26_O_4_ | 12.94 | 293.1755 | NEG | 100932311 |
| 384 | Crotaleschenine | C_16_H_23_NO_5_ | 7.01 | 274.1432 | POS | 21573658 |
| 385 | Dehydroeffusol | C_17_H_14_O_2_ | 10.44 | 249.0917 | NEG | 5316810 |
| 386 | Meridinol | C_20_H_18_O_7_ | 9.67 | 353.1012 | POS | 11079164 |
| 387 | Peucedanol | C_14_H_16_O_5_ | 9.44 | 247.0938 | POS | 15296614 |
| 388 | Loureiriol | C_16_H_14_O_6_ | 8.74 | 303.0857 | POS | 11722425 |
| 389 | Pterosin C | C_14_H_18_O_3_ | 9.36 | 279.1234 | NEG | 186209 |
| 390 | Dihydrophaseic acid | C_15_H_22_O_5_ | 7.69 | 300.1799 | POS | 11988272 |
| 391 | Purpurogallin | C_11_H_8_O_5_ | 6.09 | 221.0435 | POS | 135403797 |
| 392 | Sec-O-Glucosylhamaudol | C_21_H_26_O_10_ | 9.30 | 439.1589 | POS | 10478277 |
| 393 | 27-O-acetyl-withaferin A | C_31_H_42_O_6_ | 12.16 | 552.3305 | POS | - |
| 394 | Neolancerin | C_19_H_18_O_10_ | 8.09 | 451.0877 | NEG | 92029590 |
| 395 | Sanggenone K | C_30_H_32_O_6_ | 11.02 | 471.2190 | POS | 44258299 |
| 396 | Cleroindicin F | C_8_H_10_O_3_ | 6.50 | 119.0494 | POS | 10374646 |
| 397 | Irilone | C_16_H_10_O_6_ | 8.94 | 343.0453 | NEG | 5281779 |
| 398 | 8-Acetyl-7-Hydroxycoumarin | C_11_H_8_O_4_ | 5.20 | 249.0399 | NEG | 5411574 |
| 399 | Isofraxidin | C_11_H_10_O_5_ | 8.79 | 264.0861 | POS | 5318565 |
| 400 | 2,4-Dodecadienoic acid isobutylamide | C_16_H_29_NO | 13.30 | 296.1968 | POS | - |
| 401 | Cyclo(Tyr-Gly) | C_11_H_12_N_2_O_3_ | 5.04 | 221.0918 | POS | 138604 |
| 402 | Esculin | C_15_H_16_O_9_ | 6.27 | 339.0716 | NEG | 5281417 |
| 403 | Hydroprotopine | C_20_H_20_NO_5_^+^ | 9.00 | 377.1200 | POS | - |
| 404 | 2-(-D-Glucopyranosyloxy)-4-hydroxybenzenepropanoi | C_15_H_20_O_9_ | 8.35 | 309.0962 | POS | - |
| 405 | threo-1-C-Syringylglycerol | C_11_H_16_O_6_ | 6.46 | 227.0910 | POS | 75492726 |
| 406 | 5-Hydroxy-7-acetoxyflavone | C_17_H_12_O_5_ | 8.75 | 341.0661 | NEG | 5420895 |
| 407 | rac-Glycerol 1-myristate | C_17_H_34_O_4_ | 13.42 | 323.2199 | NEG | 79050 |
| 408 | Securiterpenoside | C_11_H_18_O_8_ | 1.15 | 296.1334 | POS | 5321222 |
| 409 | trans-Dehydrocurvularin | C_16_H_18_O_5_ | 6.53 | 271.0973 | NEG | 6438143 |
| 410 | Erigeroside | C_11_H_14_O_8_ | 5.45 | 255.0506 | NEG | 162876 |
| 411 | 4''-methyloxy-Genistin | C_22_H_22_O_1__0_ | 8.82 | 445.1135 | NEG | 71621984 |
| 412 | Lupinalbin A | C_15_H_8_O_6_ | 10.24 | 329.0299 | NEG | 5324349 |
| 413 | Perilloside B | C_16_H_24_O_7_ | 11.14 | 365.1024 | NEG | - |
| 414 | luteolin | C_15_H_10_O_6_ | 10.55 | 287.0545 | POS | 5280445 |
| 415 | Rhodiocyanoside A | C_11_H_17_NO_6_ | 1.08 | 301.1388 | POS | 6442274 |
| 416 | N-Vanillyldecanamide | C_18_H_29_NO_3_ | 10.20 | 306.2070 | NEG | 169252 |
| 417 | Axillarin | C_17_H_14_O_8_ | 8.23 | 327.0505 | NEG | 5281603 |
| 418 | Norwogonin-8-O-glucuronide | C_21_H_18_O_1__1_ | 11.46 | 427.0659 | NEG | 14180785 |
| 419 | 5-Hydroxy-6,7-dimethoxylflavone | C_17_H_14_O_5_ | 8.95 | 331.1169 | POS | 471722 |
| 420 | 1_1_alpha,1_2_alpha-Epoxy-_3_beta,23-dihydroxy-30-norolean-20(29)-en-28,1_3_beta-olide | C_29_H_42_O_6_ | 11.94 | 504.3311 | POS | 70698158 |
| 421 | Quinaldic acid | C_10_H_7_NO_2_ | 7.04 | 215.0814 | POS | 7124 |
| 422 | _5_alpha-Hydroxychloranthalactone A | C_15_H_16_O_3_ | 7.75 | 286.1431 | POS | 131857139 |
| 423 | Brevifolincarboxylic acid | C_13_H_8_O_8_ | 7.24 | 293.0286 | POS | 9838995 |
| 424 | Artemitin | C_20_H_20_O_8_ | 8.41 | 389.1200 | POS | 5320351 |
| 425 | Naringenin-7-O-beta-D-glucuronide | C_21_H_20_O_11_ | 8.23 | 449.1070 | POS | 15540754 |
| 426 | Virginiaebutanolide A | C_12_H_22_O_4_ | 11.23 | 229.1439 | NEG | 40561590 |
| 427 | Cirsimaritin | C_17_H_14_O_6_ | 8.82 | 297.0747 | POS | 188323 |
| 428 | Isovitexin | C_21_H_20_O_10_ | 8.75 | 431.0978 | NEG | 162350 |
| 429 | Piceoside | C_14_H_18_O_7_ | 9.00 | 281.1014 | POS | 92123 |
| 430 | 6(_1_H)-Azulenone, 2,3-dihydro-1,4-dimethyl | C_12_H_14_O | 10.48 | 219.1020 | NEG | 102004679 |
| 431 | Monotropein | C_16_H_22_O_11_ | 6.28 | 413.1067 | POS | 73466 |
| 432 | Indican | C_14_H_16_NO_6_ | 5.70 | 339.0679 | POS | 441564 |
| 433 | 3,7-O-Diacetylpinobanksin | C_19_H_16_O_7_ | 8.86 | 357.0959 | POS | 91884891 |
| 434 | Gastrodin | C_13_H_18_O_7_ | 6.59 | 309.0940 | POS | 115067 |
| 435 | 3,5-DIHYDROXY-4-METHOXYBENZOIC ACID | C_8_H_8_O_5_ | 8.06 | 183.0290 | NEG | - |
| 436 | Secoxyloganin methyl ester | C_18_H_26_O_11_ | 8.14 | 383.1329 | POS | 14105070 |
| 437 | Zeatin | C_10_H_13_N_5O_ | 6.61 | 264.0838 | POS | 449093 |
| 438 | Mirificin | C_26_H_28_O_13_ | 9.48 | 547.1454 | NEG | 21676217 |
| 439 | Diderroside | C_19_H_28_O_13_ | 8.94 | 501.1034 | NEG | 23760099 |
| 440 | Kaempferol-3-O-galactoside | C_21_H_20_O_11_ | 9.08 | 447.0928 | NEG | 5282149 |
| 441 | Damnacanthol | C_16_H_12_O_5_ | 7.83 | 317.1013 | POS | - |
| 442 | Altholactone | C_13_H_12_O_4_ | 8.94 | 277.0713 | NEG | 442513 |
| 443 | Jaceosidin | C_17_H_14_O_7_ | 11.13 | 329.0662 | NEG | 5379096 |
| 444 | N-Acetyl-Neuraminic Acid | C_11_H_19_NO_9_ | 1.67 | 290.0877 | NEG | 439197 |
| 445 | cafestol | C_20_H_28_O_3_ | 12.38 | 299.1975 | POS | 108052 |
| 446 | Cyclo(Tyr-Hpro) | C_14_H_16_N_2_O_4_ | 7.68 | 241.0968 | POS | 102004913 |
| 447 | Pantoyllactone glucoside | C_12_H_20_O_8_ | 7.68 | 257.1014 | POS | 14701560 |
| 448 | Demethylwedelolactone | C_15_H_8_O_7_ | 9.04 | 299.0192 | NEG | 5489605 |
| 449 | Hispidulin | C_16_H_12_O_6_ | 10.48 | 301.0702 | POS | 5281628 |
| 450 | Isoquercitrin | C_21_H_20_O_12_ | 9.01 | 463.0878 | NEG | 5280804 |
| 451 | Plumieride | C_21_H_26_O_12_ | 8.86 | 491.1189 | NEG | 72319 |
| 452 | Jasminoside N | C_22_H_38_O_11_ | 10.60 | 501.2297 | POS | 101505270 |
| 453 | Xanthotoxol | C_11_H_6_O_4_ | 5.23 | 247.0242 | NEG | 65090 |
| 454 | Deoxyinosine | C_10_H_11_N_4_O_4_ | 4.45 | 269.1126 | POS | 135398593 |
| 455 | L-LYSINE | C_6_H_14_N_2_O_2_ | 0.77 | 147.1127 | POS | 5962 |
| 456 | Skimmin | C_15_H_16_O_8_ | 8.35 | 325.0910 | POS | 99693 |
| 457 | _8_alpha-Hydroxyhirsutinolide | C_15_H_20_O_6_ | 12.11 | 261.1118 | POS | 70690654 |
| 458 | 3,19-Dihydroxy-6,23-dioxo-12-ursen-28-oic acid | C_30_H_44_O_6_ | 12.15 | 518.3467 | POS | 15460490 |
| 459 | Jasmonic acid | C_12_H_18_O_3_ | 8.54 | 252.1591 | POS | 5281166 |
| 460 | Astraganoside | C_23_H_28_O_11_ | 10.77 | 481.1695 | POS | 134715187 |
| 461 | Quercetin 3-O-glucuronide | C_21_H_18_O_13_ | 9.04 | 477.0669 | NEG | 5274585 |
| 462 | 10-O-Coumaroyl-10-O-deacetylasperuloside | C_25_H_26_O_12_ | 8.04 | 563.1403 | NEG | 95224286 |
| 463 | 2-O-Methyluridine | C_10_H_14_N_2_O_6_ | 0.99 | 291.1181 | POS | 102212 |
| 464 | Alizarin | C_14_H_8_O_4_ | 7.47 | 285.0404 | NEG | 6293 |
| 465 | (-)-Epigallocatechin | C_15_H_14_O_7_ | 5.19 | 305.0663 | NEG | 10425234 |
| 466 | Axillarin 4'-glucuronide | C_23_H_22_O_1_4 | 7.48 | 503.0830 | NEG | - |
| 467 | Parthenocissin A | C_28_H_22_O_6_ | 10.53 | 455.1480 | POS | - |
| 468 | Myricananin A | C_20_H_24_O_5_ | 9.20 | 389.1334 | POS | 25141365 |
| 469 | quercetin | C_15_H_10_O_7_ | 8.69 | 303.0493 | POS | 5280343 |
| 470 | Isorhamnetin 3-glucuronide | C_22_H_20_O_13_ | 7.99 | 491.0826 | NEG | 5491630 |
| 471 | Rhodiosin | C_27_H_30_O_16_ | 7.49 | 633.1415 | POS | - |
| 472 | Diacetylpiptocarphol | C_19_H_24_O_9_ | 9.68 | 397.1485 | POS | 102004563 |
| 473 | Licofuranocoumarin | C_21_H_20_O_7_ | 9.83 | 429.1185 | NEG | 5319001 |
| 474 | Iridin | C_24_H_26_O_13_ | 9.87 | 523.1437 | POS | 5281777 |
| 475 | 3,4-Dicaffeoylquinic acid | C_25_H_24_O_12_ | 8.94 | 515.1189 | NEG | 5281780 |
| 476 | Ferulamide | C_10_H_11_NO_3_ | 6.19 | 238.0716 | NEG | 6433734 |
| 477 | Ampelopsin A | C_28_H_22_O_7_ | 11.48 | 453.1323 | POS | - |
| 478 | Viscumneoside III | C_27_H_32_O_15_ | 8.69 | 561.1594 | POS | 195287 |
| 479 | Diosmetin | C_16_H_12_O_6_ | 9.39 | 301.0697 | POS | 5281612 |
| 480 | Isoschaftoside | C_26_H_28_O_14_ | 8.54 | 563.1403 | NEG | 3084995 |
| 481 | Koenigine | C_19_H_18_NO_3_ | 9.85 | 329.1026 | NEG | 5318825 |
| 482 | Grasshopper ketone | C_13_H_20_O_3_ | 8.39 | 205.1227 | NEG | 10220146 |
| 483 | Verproside | C_22_H_26_O_1_3 | 8.85 | 463.1225 | POS | 12000799 |
| 484 | Taxifoliol | C_15_H_12_O_7_ | 5.23 | 303.0506 | NEG | 439533 |
| 485 | Scutebarbatine Z | C_26_H_33_NO_5_ | 8.63 | 484.2068 | POS | 46929396 |
| 486 | Odontoside | C_20_H_22_O_11_ | 9.44 | 403.1015 | POS | 5320735 |
| 487 | Caftaric acid | C_13_H_12_O_9_ | 7.12 | 277.0338 | POS | 6440397 |
| 488 | Juglanin | C_20_H_18_O_10_ | 8.69 | 417.0823 | NEG | 5318717 |
| 489 | Quercimeritrin | C_21_H_20_O_12_ | 7.66 | 463.0878 | NEG | 5282160 |
| 490 | Rubrofusarin-6-O-beta-D-gentiobioside | C_27_H_32_O_15_ | 8.71 | 577.1559 | NEG | 503733 |
| 491 | 4',5,7-Trihydroxy 3,3',6,8-tetramethoxyflavone | C_19_H_18_O_9_ | 7.60 | 355.0803 | POS | 5386959 |
| 492 | Granatomycin E | C_22_H_22_O_11_ | 9.38 | 461.1086 | NEG | 132331203 |
| 493 | Lupinol C | C_20_H_18_O_7_ | 8.82 | 393.0959 | POS | 24094131 |
| 494 | p-Vinylphenyl O-[beta-D-apiofuranosyl-(1-6)]-beta-D-glucopyranoside | C_19_H_26_O_10_ | 8.79 | 395.1342 | NEG | 91895375 |
| 495 | Vitexin 2''-O-beta-D-glucoside | C_27_H_30_O_15_ | 7.28 | 595.1650 | POS | 5280641 |
| 496 | Scutellarin methyl ester | C_22_H_20_O_12_ | 7.37 | 475.0876 | NEG | 14162695 |
| 497 | Luteolin-3-O-beta-D-glucuronide | C_21_H_18_O_12_ | 8.87 | 461.0724 | NEG | 10253785 |
| 498 | Huperzine A | C_15_H_18_N_2_O | 9.10 | 241.1339 | NEG | 854026 |
| 499 | 2-Ethyl-3-methylmaleimide N-alpha-D-glucopyranoside | C_13_H_19_NO_7_ | 0.88 | 319.1493 | POS | - |
| 500 | kaempferol | C_15_H_10_O_6_ | 9.66 | 287.0544 | POS | 5280863 |
| 501 | EURYCOMANONE | C_20_H_24_O_9_ | 12.66 | 373.1273 | POS | 13936691 |
| 502 | Luteolin 7-glucuronide | C_21_H_18_O_12_ | 9.51 | 461.0721 | NEG | 5282153 |
| 503 | Glycyrrhiza flavonol A | C_20_H_18_O_7_ | 9.35 | 415.1028 | NEG | 5317765 |
| 504 | Methylmalonic acid | C_4_H_6_O_4_ | 5.22 | 278.0877 | POS | 487 |
| 505 | toralactone | C_15_H_12_O_5_ | 12.50 | 271.0609 | NEG | 5321980 |
| 506 | visamminol-3'-O- glucoside | C_21_H_26_O_10_ | 9.86 | 437.1447 | NEG | 163358503 |
| 507 | Dihydrocarpanone | C_20_H_20_O_6_ | 14.19 | 357.1324 | POS | - |
| 508 | Eupatorin | C_18_H_16_O_7_ | 12.35 | 345.0964 | POS | 97214 |
| 509 | Moracin P | C_19_H_18_O_5_ | 12.89 | 371.1139 | NEG | 25208124 |
| 510 | Protocetraric acid | C_18_H_14_O_9_ | 10.67 | 357.0595 | POS | 5489486 |
| 511 | Jaborosalactone D | C_28_H_40_O_6_ | 10.89 | 490.3154 | POS | 268947 |
| 512 | Niazirin | C_14_H_17_NO_5_ | 9.57 | 302.0992 | POS | 129556 |
| 513 | Cedrin | C_16_H_14_O_8_ | 9.90 | 317.0648 | POS | 21721881 |
| 514 | Syringin | C_17_H_24_O_9_ | 7.37 | 371.1342 | NEG | 5316860 |
| 515 | 1-(3,4,5-Trihydroxypentanoyl)-alpha-carboline | C_16_H_15_N_2_O_4_ | 6.89 | 363.1177 | POS | - |
| 516 | N-(1-Carboxy-2-phenylethyl)glutamine | C_14_H_18_N_2_O_5_ | 6.53 | 293.1140 | NEG | 558649 |
| 517 | resveratrol | C_14_H_12_O_3_ | 8.99 | 227.0707 | NEG | 445154 |
| 518 | Dihydrolycorine | C_16_H_19_NO_4_ | 6.18 | 290.1380 | POS | - |
| 519 | 3,7-Di-O-methylducheside A | C_22_H_20_O_12_ | 8.24 | 511.0634 | NEG | - |
| 520 | (E)-6-O-(p-coumaroyl)scandoside methyl ester | C_26_H_30_O_13_ | 7.06 | 595.1666 | NEG | 145874216 |
| 521 | Feruloylputrescine | C_14_H_20_N_2_O_3_ | 6.27 | 263.1397 | NEG | 5281796 |
| 522 | Aurantio-obtusin Beta-D-glucoside | C_23_H_24_O_12_ | 9.37 | 493.1334 | POS | 442725 |
