# Supplementary material for: Fracture-healing effects of Rhizoma Musae ethanolic extract: An integrated study using UHPLC-Q-Exactive-MS/MS, network pharmacology, and molecular docking
Source: PLoS One. 2025 Jan 14;20(1):e0313743. doi: 10.1371/journal.pone.0313743 (PMC11731732; doi:10.1371/journal.pone.0313743)
Supplement: S2 Table — (DOCX) [file pone.0313743.s002.docx]

**S2 Table. Analysis of the topological parameters of the core targets.**

| Target | Degree | BetweennessCentrality | ClosenessCentrality |
| --- | --- | --- | --- |
| AKT1 | 163 | 0.060431062 | 0.701265823 |
| IL6 | 153 | 0.066853301 | 0.683950617 |
| EGFR | 137 | 0.035426728 | 0.65952381 |
| STAT3 | 133 | 0.029540263 | 0.656398104 |
| CASP3 | 128 | 0.021800871 | 0.642691415 |
| HIF1A | 127 | 0.028594643 | 0.642691415 |
| SRC | 125 | 0.045036341 | 0.639722864 |
| BCL2 | 121 | 0.016691488 | 0.630979499 |
| ESR1 | 119 | 0.026902411 | 0.626696833 |
| HSP90AA1 | 118 | 0.032545877 | 0.630979499 |
| PPARG | 116 | 0.041258845 | 0.628117914 |
| MAPK3 | 115 | 0.019105192 | 0.625282167 |
| CCND1 | 105 | 0.011450507 | 0.604803493 |
| MMP9 | 104 | 0.017417705 | 0.606126915 |
| MTOR | 102 | 0.014159409 | 0.603485839 |
| PTGS2 | 102 | 0.022938889 | 0.604803493 |
| HSP90AB1 | 100 | 0.012908835 | 0.5995671 |
| ERBB2 | 98 | 0.009977239 | 0.5995671 |
| GSK3B | 96 | 0.013667446 | 0.596982759 |
| SIRT1 | 89 | 0.012294007 | 0.581932773 |
| MDM2 | 83 | 0.005928133 | 0.577083333 |
| BCL2L1 | 82 | 0.004555601 | 0.571134021 |
| MAPK1 | 82 | 0.006807712 | 0.573498965 |
| FGF2 | 78 | 0.006781426 | 0.566462168 |
| EP300 | 78 | 0.008428471 | 0.56300813 |
| JAK2 | 77 | 0.008448702 | 0.56300813 |
| APP | 76 | 0.02054511 | 0.569958848 |
| MMP2 | 74 | 0.006614947 | 0.561866126 |
| CDC42 | 74 | 0.006368903 | 0.5562249 |
| PRKACA | 72 | 0.019963175 | 0.561866126 |
| CDK4 | 69 | 0.009869284 | 0.54743083 |
| IL2 | 68 | 0.010481469 | 0.549603175 |
| HSPA5 | 66 | 0.006165385 | 0.549603175 |
| PPARA | 64 | 0.012379282 | 0.551792829 |
| CDK2 | 63 | 0.005655344 | 0.545275591 |
| NR3C1 | 62 | 0.006404941 | 0.552894212 |
| HDAC1 | 62 | 0.00531179 | 0.542074364 |
| PDGFRB | 60 | 0.009186884 | 0.543137255 |
| CYP3A4 | 59 | 0.014515354 | 0.541015625 |
| ACE | 58 | 0.006843567 | 0.549603175 |
| FASN | 58 | 0.013673363 | 0.532692308 |
| SERPINE1 | 56 | 0.008110254 | 0.536821705 |
| ABCB1 | 54 | 0.012197293 | 0.534749035 |
| HMGCR | 54 | 0.015402943 | 0.536821705 |
| MET | 52 | 0.004146346 | 0.527619048 |
| ABCG2 | 49 | 0.009016921 | 0.530651341 |
| MAPT | 49 | 0.00792585 | 0.525616698 |
| ESR2 | 46 | 0.004504529 | 0.527619048 |
| CYP19A1 | 45 | 0.006855697 | 0.524621212 |
| AGTR1 | 43 | 0.013792764 | 0.514869888 |
| CYP1A1 | 42 | 0.004852203 | 0.515828678 |
| COMT | 41 | 0.008503619 | 0.506398537 |
| RXRA | 41 | 0.005295185 | 0.512962963 |
| EIF4E | 40 | 0.009328607 | 0.499099099 |
| MAOB | 38 | 0.009034693 | 0.510128913 |
| DRD2 | 38 | 0.008784175 | 0.507326007 |
| AURKA | 38 | 0.004280013 | 0.490265487 |
| CYP1A2 | 36 | 0.00498202 | 0.504553734 |
| ADRB2 | 35 | 0.007286319 | 0.511070111 |
