# Supplementary material for: Fracture-healing effects of Rhizoma Musae ethanolic extract: An integrated study using UHPLC-Q-Exactive-MS/MS, network pharmacology, and molecular docking
Source: PLoS One. 2025 Jan 14;20(1):e0313743. doi: 10.1371/journal.pone.0313743 (PMC11731732; doi:10.1371/journal.pone.0313743)
Supplement: S1 Graphical abstract — (PDF) [file pone.0313743.s003.pdf]

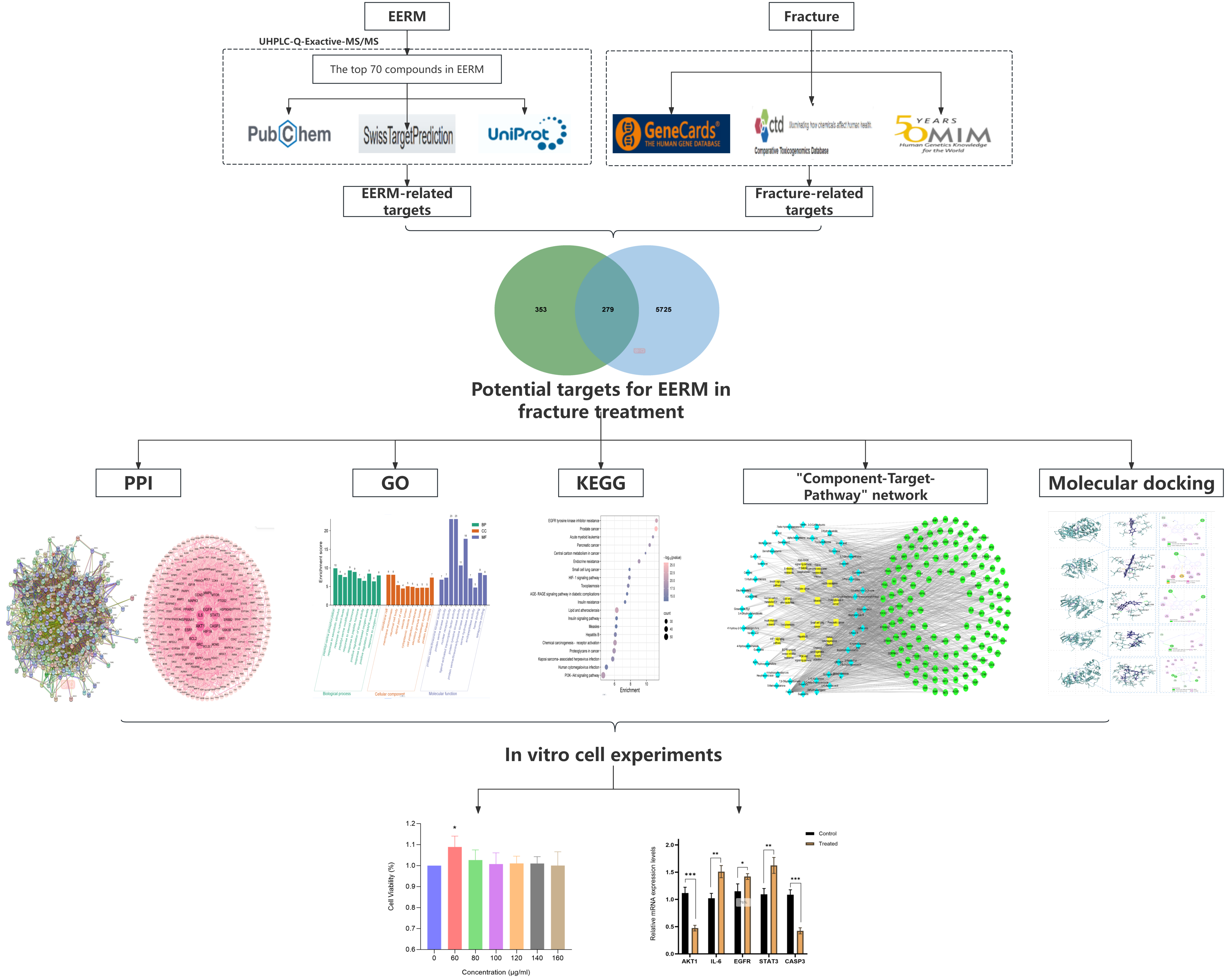

EERM
UHPLC-Q-Exactive-MS/MS
The top 70 compounds in EERM
PubChem
SwissTargetPrediction
UniProt
EERM-related targets
Fracture
GeneCards
THE HUMAN GENE DATABASE
ctd
Comparative Toxicogenomics Database
50 YEARS OMIM
Human Genetics Knowledge for the World
Fracture-related targets
353
279
5725
Potential targets for EERM in fracture treatment
PPI
GO
KEGG
"Component-Target-Pathway" network
Molecular docking
Enrichment score
Biological process
Cellular component
Molecular function
BP
CC
MF
Enrichment
In vitro cell experiments
Cell Viability (%)
Concentration (μg/ml)
Relative mRNA expression levels
Control
Treated
AKT1
IL-6
EGFR
STAT3
CASP3
